# Homologous Recombination Deficiency Unrelated to Platinum and PARP Inhibitor Response in Cell Line Libraries

**DOI:** 10.1038/s41597-024-03018-4

**Published:** 2024-02-06

**Authors:** Shiro Takamatsu, Kosuke Murakami, Noriomi Matsumura

**Affiliations:** 1 Department of Gynecology and Obstetrics, Kyoto University Graduate School of Medicine, Kyoto, Kyoto, Japan; 2 Department of Genomic Medicine, the University of Texas MD Anderson Cancer Center, Houston, Texas USA; 3 Department of Obstetrics and Gynecology, Kindai University Faculty of Medicine, Osaka-Sayama, Osaka, Japan; 4 The Bloomberg~Kimmel Institute for Cancer Immunotherapy, Johns Hopkins University, Baltimore, Maryland USA

**Keywords:** Drug development, Tumour biomarkers

## Abstract

While large publicly available cancer cell line databases are invaluable for preclinical drug discovery and biomarker development, the association between homologous recombination deficiency (HRD) and drug sensitivity in these resources remains unclear. In this study, we comprehensively analyzed molecular profiles and drug screening data from the Cancer Cell Line Encyclopedia. Unexpectedly, gene alterations in *BRCA1/2* or homologous recombination-related genes, HRD scores, or mutational signature 3 were not positively correlated with sensitivity to platinum agents or PARP inhibitors. Rather, higher HRD scores and mutational signature 3 were significantly associated with resistance to these agents in multiple assays. These findings were consistent when analyzing exclusively breast and ovarian cancer cell lines and when using data from the COSMIC Cell Line Project. Collectively, the existing data from established cancer cell lines do not reflect the expected association between HRD status and drug response to platinum agents and PARP inhibitors in clinical tumors. This discrepancy may extend to other tumor characteristics, highlighting the importance of recognizing potential limitations in cell line data for researchers.

## Introduction

Human tumor-derived models play a crucial role in cancer research and drug development. Among these models, human cancer cell lines have been extensively utilized in basic and preclinical research due to their ease of handling and low-cost availability. It has been reported that cancer cell lines robustly retain the genomic features of their original tumors^1^. The advent of next-generation sequencing has facilitated the comprehensive molecular profiling of more than 1000 representative cancer cell lines, and these datasets are available to researchers as public resources, including the Broad institute’s Cancer Cell Line Encyclopedia (CCLE)^1^ and the Sanger institute’s COSMIC Cell Line Project (CLP)^2^. In addition, in the past decade, several high-throughput drug screening studies using these cell lines have been conducted by different groups, including the Genomics of Drug Sensitivity in Cancer (GDSC)^3^, the Cancer Therapeutic Response Portal (CTRP)^4,5^, and Profiling Relative Inhibition Simultaneously in Mixtures (PRISM)^6^, Genentech Cell Line Screening Initiative (gCSI)^7^, and others. Given the open availability of these datasets and their capacity to link specific molecular features to drug sensitivities, computational and machine learning-based approaches have been actively explored in recent years for targeted drug discovery and therapeutic biomarker identification^1,2,8–10^.

The homologous recombination repair (HR) pathway is the most precise and essential DNA damage repair mechanism for DNA double-strand breaks. Dysfunction of this pathway, known as homologous recombination deficiency (HRD), often caused by inactivation of *BRCA1/2* molecules, leads to DNA damage accumulation and genomic instability that contribute to tumor development and progression. Cancer cells with HRD exhibit characteristic genomic changes referred to as the “Genomic Scar” signatures^11–13^. In ovarian and breast cancer, *BRCA1/2* mutations and genomic scar signatures are significantly associated with the sensitivity to PARP inhibitors and platinum agents^14^. Recently, their clinical utility as biomarkers has been investigated in other cancer types^15–17^.

Although various research has been conducted using the above cancer cell line databases, there have been no reports to clarify the association between HRD status and the sensitivity to PARP inhibitors or platinum drugs. Here, we examine the association between HRD status and *in vitro* drug sensitivity to these agents in the CCLE and the CLP datasets, demonstrating significant differences from that in clinical tumors. These findings highlight the limitations of drug and biomarker discovery research based on existing cell line databases and emphasize the need for caution when interpreting results obtained from such sources.

## Results

### Association between BRCA1/2 gene alterations and drug sensitivity

Genomic profiles in the CCLE cell lines and multiple drug response data, including GDSC1/2, CTRP1/2, PRISM, and gCSI, were combined, resulting in a total of 800 assays across 1182 cell lines (Tables S1, S2). This included 10 assays for platinum agent (Carboplatin 2, Cisplatin 4, Oxaliplatin 4) and 14 assays for PARP inhibitors (Olaparib 6, Talazoparib 3, Veriparib 2, Niraparib 2, Rucaparib 1). We used the area under the drug-response curve (AUC) instead of the half maximal inhibitory concentration (IC50) as a measure of drug efficacy because there were fewer outliers and missing data for AUC than for IC50.

**Fig. 1:**
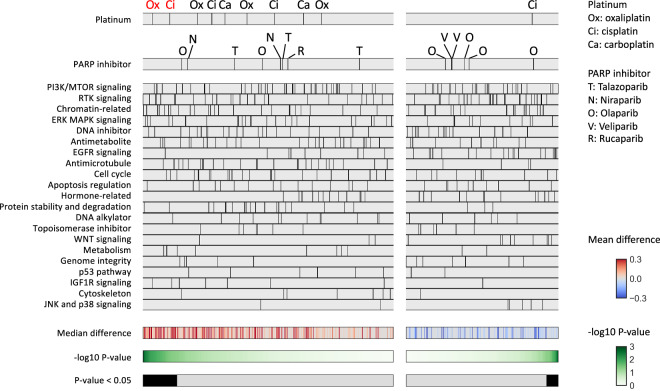
Association between *BRCA1/2* alterations and drug sensitivity. Comparison of the area under the drug-response curve (AUC) between cell lines with *BRCA1/2* alterations (n = 25) and no HR-related gene mutations (n = 872) was performed. The drugs were divided into left and right panels based on positive and negative median differences, and further ordered based on the lowest and highest p-values (Mann-Whitney test), respectively. Platinum agents (shown in the top panel) and PARP inhibitors (shown in the second panel) exhibiting a trend for positive difference (unadjusted P < 0.05) between the two groups are highlighted in red font.

We compared AUC values for each drug between cell lines with *BRCA1/2* alterations (n = 25), which include *BRCA1/2* mutations with locus-specific LOH and *BRCA1* methylations, and those without any HR-related gene mutations (n = 872) (Fig. 1). A higher AUC value of a sample indicates higher resistance to the assay. Among the 10 platinum agent assays, 2 assays (Oxaliplatin 1, Cisplatin 1) showed a trend of higher AUCs in cell lines with *BRCA* alterations (unadjusted P < 0.05, adjusted P ≧ 0.05), while no assays showed lower AUCs. Among 14 PARP inhibitor assays, none showed a significant difference between the two groups.

There was no obvious difference in drug response of platinum agents and PARP inhibitors between samples with *BRCA1* methylation (n = 6) and those with *BRCA1/2* mutations with locus-specific LOH (n = 19) (Figure S1).

### Genomic scar signatures associated with HRD status in cancer cell lines

**Fig. 2:**
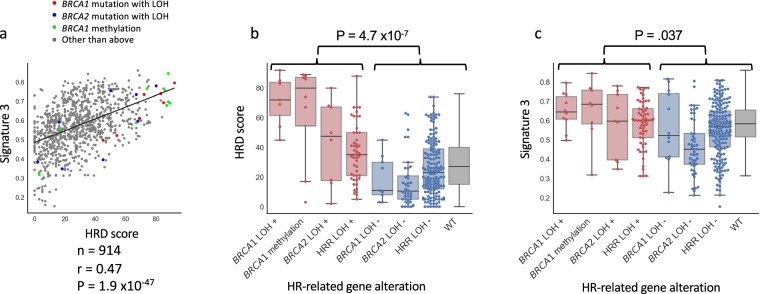
Correlations between HRD status vs genomic scar scores in the CCLE dataset. (**a**) Correlation between HRD score and mutational signature 3 and distribution of *BRCA1/2* alterations. HRD scores and mutational signature 3 values were positively correlated. (**b**) Association between HR-related gene alterations with locus-specific LOH and HRD score. HRD scores were significantly higher in samples with HR-related gene alterations the those without (Mann-Whitney test, P = 4.7 × 10^−7^). HRR LOH +/−: samples with HR-related gene mutations other than *BRCA1/2* with/without the locus-specific LOH. WT; samples without any HR-related mutations. (**c**) Association between HR-related gene alterations with locus-specific LOH and mutational signature 3. Mutational signature 3 were significantly higher in samples with HR-related gene alterations the those without (Mann-Whitney test, P = 0.037).

Genomic scar signatures, which are indicators of characteristic genomic changes associated with HRD, include the HRD score and the mutational signature 3. The HRD score quantifies the characteristic chromosomal structural changes based on the SNP array data (see methods). The mutational signature 3 is calculated from the pattern of single base substitutions in the context of flanking bases, obtained through genome-wide analysis of somatic mutational profiles. The HRD score and signature 3 were positively correlated in the CCLE dataset (n = 914, Spearman r = 0.475, p = 1.25 × 10^−52^, Fig. 2a). These two scores were significantly higher in samples with *BRCA1/2* mutation with LOH, *BRCA1* methylation, and other HR-related gene mutations with LOH compared to those with HR-related gene mutations without LOH or without any HR-related gene mutations (P = 4.7 × 10^−7^, P = 0.037 respectively, Fig. 2b,c). These findings indicate the association between tumor HRD status and genomic scar signatures, commonly observed in clinical samples, was replicated in the cancer cell lines.

In HR-related genes other than *BRCA1*, 4 genes from 6 samples (1 *RAD51C*, 1 *CDK12*, 1 *FANCF*, and 3 *RAD50*) were considered to be silenced due to DNA methylation of the promoter region, but their HRD scores and signature 3 were not significantly elevated (Figure S2). This mirrors to our previous analysis in clinical samples^16^, and their impact on HRD status was unclear.

### Association between HR-related gene alterations and drug sensitivity

**Fig. 3:**
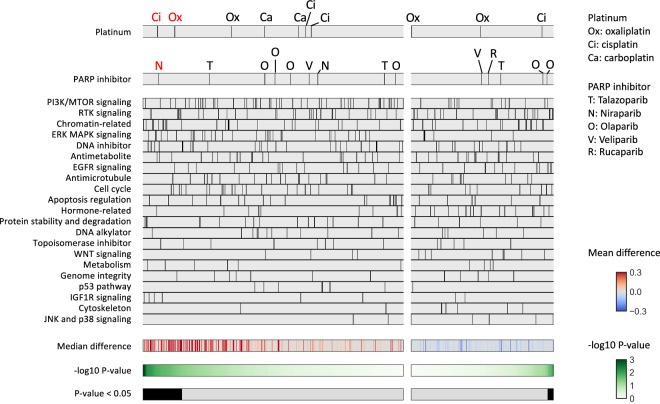
Association between HR-related gene alterations and drug sensitivity. Comparison of the area under the drug-response curve (AUC) between cell lines with HR-related gene alterations including *BRCA1/2* (n = 82) and no HR-related gene mutations (n = 872) was performed. The drugs were divided into left and right panels based on positive and negative median differences, and further ordered based on the lowest and highest p-values (Mann-Whitney test), respectively. Platinum agents (shown in the top panel) and PARP inhibitors (shown in the second panel) exhibiting a trend for positive differences (Unadjusted P < 0.05) between the two groups are highlighted in red font.

A similar comparison of AUCs for each drug was performed between cell lines with HR-related gene alterations including *BRCA1/2* (n = 82) and those without any HR-related gene alterations (n = 872) (Fig. 3). Two of the 10 platinum agents (Cisplatin 1, Oxaliplatin 1) and 1 of the 14 PARP inhibitors (Niraparib 1) showed a trend of higher AUCs in the HR-related gene-altered samples (unadjusted P < 0.05, adjusted P ≧ 0.05), and none of them showed lower AUCs. Similarly, when comparing samples with HR-related gene alterations excluding *BRCA1/2* (n = 57) to those without any HR-related gene alterations (n = 872), none of the platinum or PARP inhibitor assays showed significantly lower AUCs in the HR-related gene-altered group (Figure S3).

### Correlation between genomic scar signatures and drug sensitivity

**Fig. 4:**
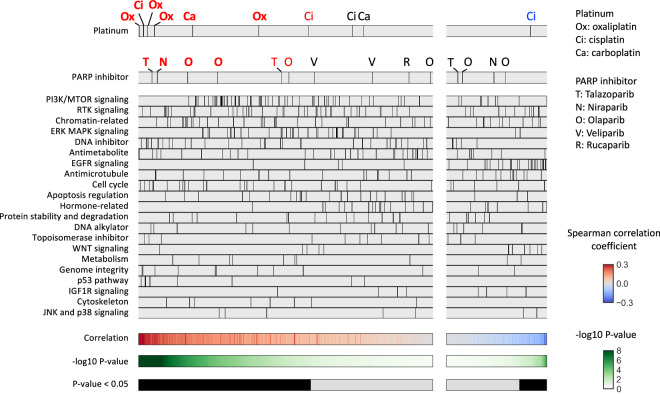
Association between HRD score and drug sensitivity. Spearman’s correlation between HRD score and the area under the drug-response curve (AUC) was analyzed. The drugs were divided into left and right panels based on positive and negative median differences, and further ordered based on the lowest and highest p-values, respectively. Platinum agents (shown in the top panel) and PARP inhibitors (shown in the second panel) exhibiting a trend for positive or negative differences (unadjusted P < 0.05) between the two groups are highlighted in red and blue font, respectively, and those remained statistically significant (adjusted P < 0.05) after multiple testing correction are indicated in bold style.

Spearman’s correlation analysis between HRD scores and AUCs (Fig. 4) showed a trend toward positive correlations in 7 and negative correlations in 1 of the 10 platinum assays (unadjusted P < 0.05). After correction for multiple testing, 6 assays (Oxaliplatin 4, Cisplatin 1, and Carboplatin 1) remained statistically significant for positive correlations (adjusted P < 0.05). Among the 14 PARP inhibitor assays, 4 (Olaparib 2, Talazoparib 1, Niraparib 1) showed positive correlations both before and after multiple testing corrections (P < 0.05), and no assays showed a trend toward negative correlations.

**Fig. 5:**
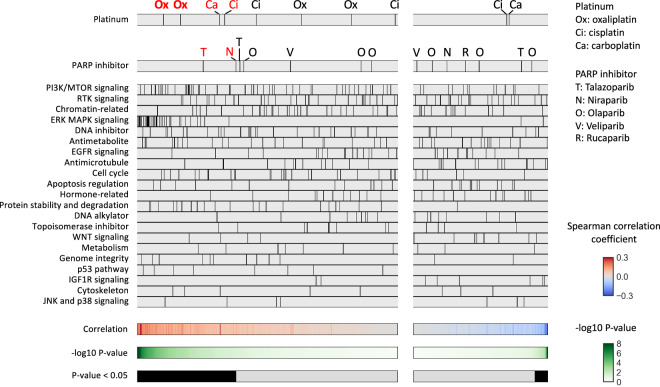
Association between signature 3 and drug sensitivity. Spearman’s correlation between mutational signature 3 value and the area under the drug-response curve (AUC) was analyzed. The drugs were divided into left and right panels based on positive and negative median differences, and further ordered based on the lowest and highest p-values, respectively. Platinum agents (shown in the top panel) and PARP inhibitors (shown in the second panel) exhibiting a trend for positive difference (unadjusted P < 0.05) between the two groups are highlighted in red font, and those remained statistically significant (adjusted P < 0.05) after multiple testing correction are indicated in bold style.

Similarly, Spearman’s correlation analysis between mutational signature 3 and AUCs (Fig. 5) showed a trend toward positive correlations in 4 of the 10 platinum assays (unadjusted P < 0.05) and 2 (Oxaliplatin 2) of them remained statistically significant (adjusted P < 0.05). No assays showed significant negative correlations. Among the 14 PARP inhibitor assays, 2 assays showed a trend toward positive correlations (unadjusted P < 0.05, adjusted P ≧ 0.05), and no assays showed a trend toward negative correlations.

Results were replicated in the same analysis restricted to breast and ovarian cancer cell lines (n = 54 and 62) (Figure S4). Furthermore, the same series of analyses using the CLP dataset, in which the molecular profiles were investigated independently of the CCLE, showed that the distributions of HRD scores and mutational signature 3 were similar in the two datasets (Figure S5), and the association with drug sensitivity was almost identical (Figure S6-S10).

These results indicate that based on analysis of the currently available cancer cell line datasets, HRD status was more likely to correlate with resistance than with susceptibility to platinum or PARP inhibitors.

## Discussion

In the current study, using the largest database of cancer cell lines, we consistently observed that HRD status was not associated with sensitivity to PARP inhibitors or platinum drugs, in contrast to data reported in clinical tumors. Many earlier experimental studies have shown that cell lines with HRD are sensitive to platinum and PARP inhibitors. Knockout of *BRCA1* or *BRCA2* in human embryonic stem cells or in the chicken B cell-derived cell line DT40 cells markedly increased sensitivity to PARP inhibitors and platinum drugs^18,19^. During cell culture of the pancreatic cancer cell line CAPAN-1 with a *BRCA2* mutation, the addition of cisplatin induced the emergence of subclones with a *BRCA2* reversion mutation, resulting in resistance to cisplatin and the PARP inhibitor AG-14361^20^. The accumulation of such experimental evidence eventually led to the clinical implementation of personalized treatment with today’s biomarker-based PARP inhibitors, and its importance is undeniable. However, it should be noted that most of these experiments examined the association between dysfunction of BRCA molecules and *in vitro* drug sensitivity in cells with identical backgrounds. In contrast, there have been limited reports investigating the association by comparing multiple cell lines derived from different backgrounds. To the best of our knowledge, only one study in 2012 reported a correlation between the TAI score, one of the genomic scar scores for HRD, and sensitivity to cisplatin in 10 breast cancer cell lines^11^. On the other hand, a study in 2020 comparing 12 breast cancer cell lines, including *BRCA* mutated lines, reported that *BRCA* status was independent of the response to PARP inhibitors^21^. Large-scale, high-throughput screens databases for cancer cell lines from diverse backgrounds may have fundamental difficulties in reproducing clinical tumor sensitivities.

The discrepancy in drug response between cancer cell lines and clinical tumors observed here can be attributed to several factors. Firstly, clinical tumor tissues are composed of complex structures that includes not only tumor cells but also mesenchymal cells and immune cells, while cancer cell lines lack such a tumor microenvironment. Recent studies of the tumor microenvironment, particularly focusing on cancer-associated fibroblasts, have accumulated evidence that plastic and dynamic interactions involving hypoxia, metabolic abnormalities, and tumor immune responses within this environment play a critical role in tumor progression as well as drug sensitivity and resistance^22^. Secondly, the *in vitro* drug screening assays examined in the cell line databases evaluated drug response over a very short period of 3–5 days, which is considerably shorter than the assessment of efficacy *in vivo* or in clinical tumors, where it is usually assessed on a weekly or monthly basis. In a report using patient-derived xenografts (PDX), it took at least 10 days for platinum to achieve effective tumor suppression in HRD tumors^23^. This suggests that differences between *in vitro* and *in vivo* assays may contribute to the observed discrepancy. In recent drug discovery methodologies, it has become increasingly important to evaluate the effect of a drug in the context of the regulation of specific signaling cascades, rather than simply cell viability^24,25^. To our knowledge, there are currently no data on such HR functional assays across multiple cancer cell lines, but such data may shed light on this issue in the future. Thirdly, the process of establishing immortalized cell lines from tumor tissues inevitably involves significant selection pressure. During this process, fast-proliferating clones may dominate the heterogeneous cell population of a tumor, or clones that have acquired advantageous traits for growth on 2D plates through gene mutations during cell culture may be preferentially selected^26^. It has been reported that disruption of the HR pathway in cells, such as through knockout of the *BRCA1/2* genes, leads to suppression of cell proliferation^27,28^. This is likely because cells deficient in HR function use alternative DNA repair pathways to repair DNA damage while the cell cycle checkpoint is activated and proliferation is halted. However, given intratumor heterogeneity, when attempting to establish cancer cell lines from tumors with HRD, slow-growing cells may be eliminated and fast-growing cells with highly enhanced alternative mechanisms may be selected. Consequently, the established cell line library may possess different properties compared to clinical tumors.

It has long been well documented that *in vivo* drug studies using PDX models more closely resemble clinical tumors than *in vitro* studies with cancer cell lines^29,30^. Specifically, in the context of HRD and PARP inhibitors, the tumor shrinkage effect of niraparib treatment on ovarian cancer PDX models was greater in tumors with *BRCA* mutations and higher HRD scores^31^. The National Cancer Institute has performed comprehensive molecular characterization of a large number of human tumors, their corresponding PDX models, and subsequent passages in various types of cancer using next-generation sequencing, and the results have been published in the Patient-Derived Models Repository (PDMR) database^32^. The addition of drug sensitivity data to these resources holds significant promise for advancing cancer drug research in the future. In addition, 3D *in vitro* cultures such as patient-derived organoids have been utilized in recent studies because they behave more like clinical tumors than 2D cell lines and can be cultivated with higher efficiency than PDX models^33,34^. However, it has been reported that even in organoids, drug sensitivity may or may not accurately reflect that of clinical tumors, depending on the culture medium and the type of drug^35^. Therefore, careful consideration of experimental conditions is required to ensure accurate evaluation of drug responses in organoids.

In conclusion, we analyzed molecular characteristics and drug screening data from the largest currently available cancer cell line library and found that differences in HRD status between cell lines do not correlate with sensitivity to platinum or PARP inhibitors in 2D cell culture assays. This critical deviation from the anticipated findings in clinical oncology may also apply to molecularly-targeted drugs other than PARP inhibitors, warranting caution among researchers using the database for future cancer research.

## Methods

### Cancer cell line datasets

The gene mutations, copy number aberrations, gene expression, and Reduced Representation Bisulfite Sequencing (RRBS)-based DNA methylation profiles for the Cancer Cell Line Encyclopedia (CCLE) dataset were obtained from the Dependency Map (DepMap) portal (Table S1).

For the COSMIC-Cell Line Project (CLP) dataset, the gene mutations, copy number aberrations, gene expression, and aneuploidy data were downloaded from the COSMIC website (GRCh37, release v97, 29th November 2022). In addition, the DNA methylation array data for these cell lines were obtained from the Gene Expression Omnibus (GEO) database (GSE68379)^2,36^. (Table S1).

### HR-related gene mutations with locus-specific LOH

Based on a previous report^37^, 29 genes were defined as HR-related genes: *ATM, ATR, BARD1, BLM, BRCA1, BRCA2, BRIP1, CDK12, CHEK1, CHEK2, FANCA, FANCC, FANCD2, FANCE, FANCF, FANCI, FANCL, FANCM, MRE11, NBN, PALB2, RAD50, RAD51, RAD51B, RAD51C, RAD51D, RAD52, RAD54L*, and *RPA1*.

From the CCLE gene mutation profiles, mutations with “Variant_annotation” of “damaging” were obtained. From the CLP mutation profile, truncating mutations and missense mutations that were annotated as “deleterious” or “damaging” in all of 10 functional impact prediction methods (SIFT, Polyphen2, LRT, MutationTaster, MutationAssessor, FATHMM, PROVEAN, MetaSVM, MetaLR, M-CAP) were retained.

As described in a previous report^16^, the estimated copy number of the minor allele at the locus of each mutation was evaluated. A value of 0 indicated the presence of LOH, while a value of 1 or more indicated the absence of LOH. Mutations with unknown locus-specific copy numbers in either the CCLE or CLP dataset were determined for LOH status using the other dataset. Samples with mutations that still had unknown LOH were excluded from the relevant analysis.

### Mutational signature 3

Using all SNVs extracted from gene mutation profiles as input, the cosine similarity to the mutational signature 3 of COSMIC version 2 reference signatures was calculated using SigMA^38^.

### HRD score

The scarHRD^39^ was utilized to calculate the telomeric allelic imbalance (TAI)^11^, large-scale state transition (LST)^12^, and genomic loss of heterozygosity (LOH) scores^13^ based on the segmented allele-specific copy number data. The HRD score was determined as the sum of these scores.

### BRCA1 methylation

Gene expression and methylation beta values of the *BRCA1* promoter regions were analyzed to annotate methylation silencing for each cell line. Specifically, in the CCLE dataset, samples with a beta value > 0.3 in two promoter regions located 1Kb upstream from the transcription start site and gene expression < 10% were considered to have *BRCA1* methylation (Figure S11a). In the CLP dataset, significant negative correlations were observed between beta values and *BRCA1* gene expression in 17 promoter methylation probes. Samples with beta values > 0.2 and gene expression < 30% for at least 15 of the 17 probes were considered to have *BRCA1* methylation (Figure S11b).

### Drug sensitivity datasets

Drug sensitivity screening data for GDSC1/2^3^, CTRP1/2^4,5^, and PRISM^6^ were obtained from their original websites (Table S2). For the gCSI dataset^7^, curated drug response data were obtained from PharmacoDB^40,41^. We employed the area under the drug-response curve (AUC) values rather than the half maximal inhibitory concentration (IC50) for comparing drug effects, because the AUC reportedly contains fewer outliers compared to the IC50^42^, and, indeed, had fewer missing values in the datasets. In these datasets, the same drug may have been assayed multiple times under different conditions. For an unbiased evaluation, we analyzed all such assays separately without any modification. Among the compounds in the CTRP1/2, those with clinical application or FDA approval were retained. Similarly, among the compounds studied in the PRISM, those relevant to the field of oncology were retained. All drugs were integrated using their drug name and PubChem compound ID, and then categorized into 24 classes based on drug annotations in the GDSC and CTRP. The categories included Platinum, PARP inhibitor, Topoisomerase inhibitor, DNA alkylator, DNA inhibitor, Antimetabolite, PI3K/MTOR signaling, RTK signaling, Chromatin-related, ERK MAPK signaling, Cell cycle-related, Anti-microtubular, Protein stability and degradation, Apoptosis regulation, EGFR signaling, WNT signaling, Genome integrity, Metabolism, Hormone-related, IGF1R signaling, p53 pathway, Cytoskeleton, JNK and p38 signaling, and Others (Table S3). Assays with insufficient number of samples for comparison were excluded; in the CCLE datasets, 82 assays with fewer than 10 samples with HR-related gene alterations were excluded and the remaining 800 assays were analyzed; in the CLP datasets, 213 assays with fewer than 10 samples with HR-related gene alterations were excluded and the remaining 669 assays were analyzed.

### Statistical analysis

All statistical analyses were performed using Python (3.8.8). Comparison of drug sensitivity between cell lines per assay was performed by the Mann-Whitney and Spearman’s correlation tests using SciPy module (1.7.2). P values were corrected for multiple testing using the Holm method. Adjusted P value < 0.05 was considered statistically significant.

### Ethics

This study used only previously published anonymized data and was exempt from institutional review board approvals and informed consent for patients.

### Supplementary information

Supplementary Information

## Data Availability

All the data and codes used in this study has been uploaded on the Figshare page^43^. This dataset contains integrated multi-omic profiles of cancer cell lines from the CCLE and COSMIC-CLP projects and their drug response data with category annotation from the GDSC1/2, CTRPv1/v2, PRISM, and gCSI studies.

## Code availability

Codes to reproduce the results of this work are available on the Figshare^43^ and the GitHub project page (https://github.com/shirotak/CellLine_HRD_DrugRes).
