# Supplementary material for: Homologous Recombination Deficiency Unrelated to Platinum and PARP Inhibitor Response in Cell Line Libraries
Source: Sci Data. 2024 Feb 6;11:171. doi: 10.1038/s41597-024-03018-4 (PMC10847511; doi:10.1038/s41597-024-03018-4)
Supplement: Supplementary file 1 — Supplementary Information [file 41597_2024_3018_MOESM1_ESM.pdf]

Supplementary Information

Supplementary Figures

Figure S1) Comparison of AUC values in platinum agents and PARP inhibitors between samples with BRCA1 methylation (n=6) and samples with BRCA1/2 mutations with locus-specific LOH (n=19)
Figure S2) Analysis of methylation silencing in HR-related genes other than BRCA1
Figure S3) Association between HR-related gene alterations excluding BRCA1/2 and drug sensitivity
Figure S4) Analyses in breast (n=54) and ovarian (n=62) cell lines in the CCLE dataset
Figure S5) The distributions of HRD scores and mutational signature 3 between CCLE and CLP datasets
Figure S6) Association between BRCA1/2 alterations and drug sensitivity in the CLP dataset
Figure S7) Association between HR-related gene alterations and drug sensitivity in the CLP dataset
Figure S8) Correlations between HRD status vs genomic scar scores in the CLP dataset
Figure S9) Association between HRD score and drug sensitivity in the CLP dataset
Figure S10) Association between signature 3 and drug sensitivity in the CLP dataset
Figure S11) Analysis of methylation silencing in BRCA1

Supplementary Tables

Table S1) Sources for molecular profiling of cancer cell lines
Table S2) Sources of drug response data
Table S3) Drug category annotations for each drug Integrated across datasets (GDSC, CTRP, PRISM, gCSI)

Figure S1) Comparison of AUC values in platinum agents and PARP inhibitors between samples with BRCA1 methylation (n=6) and samples with BRCA1/2 mutations with locus-specific LOH (n=19)

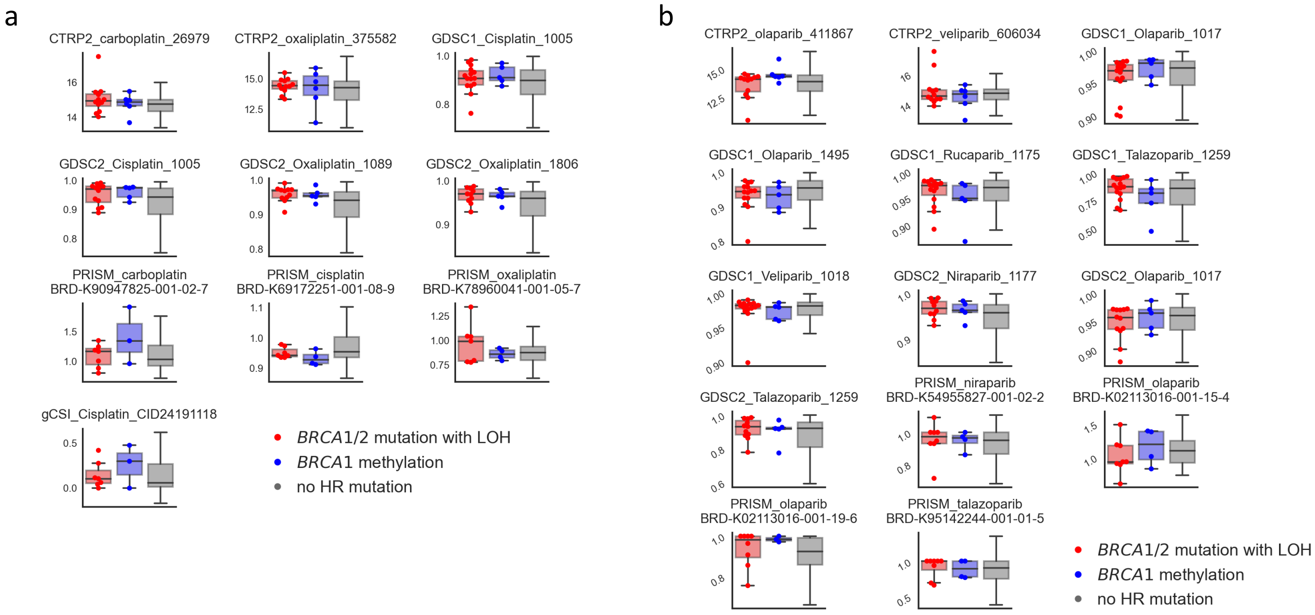

a) Comparison in platinum agents.
   No assays showed significant differences between the BRCA1 methylation and BRCA1/2 mutation groups. (CTRP2_carboplatin_26979; P = 0.673, CTRP2_oxaliplatin_375582; P = 1.0, GDSC1_Cisplatin_1005; P = 0.866, GDSC2_Cisplatin_1005; P = 0.879, GDSC2_Oxaliplatin_1089; P = 0.743, GDSC2_Oxaliplatin_1806; P = 0.768, PRISM_carboplatin_BRD-K90947825-001-02-7; P = 0.383, PRISM_cisplatin_BRD-K69172251-001-08-9; P = 0.23, PRISM_oxaliplatin_BRD-K78960041-001-05-7; P = 0.648, gCSI_Cisplatin_CID24191118; P = 0.667, Mann-Whitney test)

b) Comparison in PARP inhibitors.
   No assays showed significant differences between the BRCA1 methylation and BRCA1/2 mutation groups.
   (CTRP2_olaparib_411867; P = 0.075, CTRP2_veliparib_606034; P = 0.75, GDSC1_Olaparib_1017; P = 0.266, GDSC1_Olaparib_1495; P = 0.622, GDSC1_Rucaparib_1175; P = 0.306, GDSC1_Talazoparib_1259; P = 0.168, GDSC1_Veliparib_1018; P = 0.445, GDSC2_Niraparib_1177; P = 0.859, GDSC2_Olaparib_1017; P = 0.574, GDSC2_Talazoparib_1259; P = 0.661, PRISM_niraparib_BRD-K54955827-001-02-2; P = 0.932, PRISM_olaparib_BRD-K02113016-001-15-4; P = 0.57, PRISM_olaparib_BRD-K02113016-001-19-6; P = 0.748, PRISM_talazoparib_BRD-K95142244-001-01-5; P = 0.925, Mann-Whitney test)

Figure S2) Analysis of methylation silencing in HR-related genes other than BRCA1

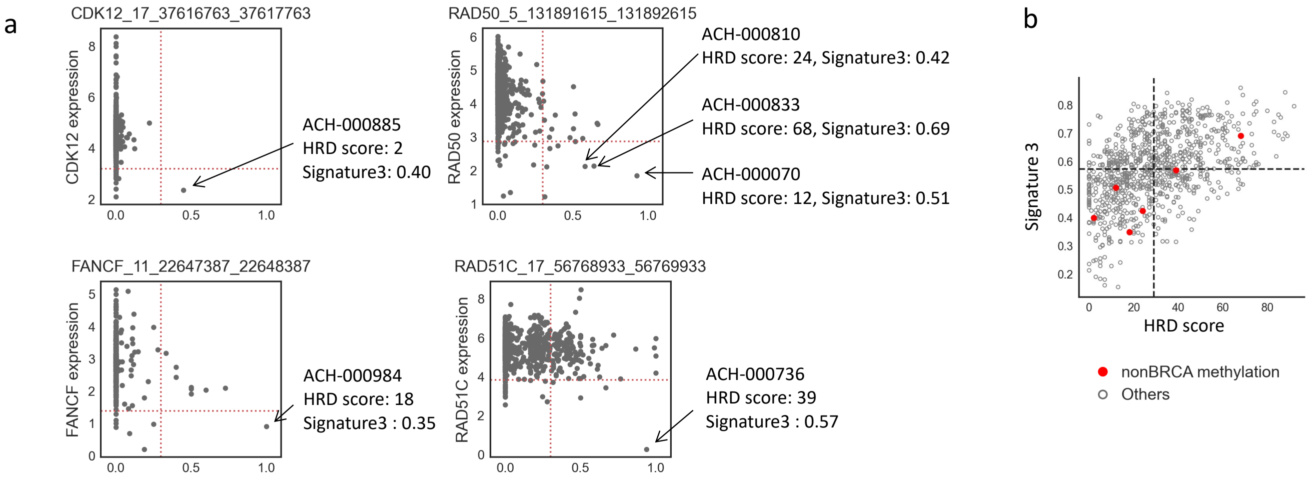

a) Identification of samples with methylation samples.
From methylation beta values of the gene promoter regions (located 1Kb upstream from the transcription start site) and gene expression (Y-axis), we selected the regions of i) mean beta value < 0.3, ii) Spearman correlation between beta value and gene expression r < 0 and p < 0.05. After drawing scatter plots for the four selected regions, samples with extremely higher methylation and lower gene expression than the other samples were visually identified and determined to be methylation cases.
4 genes from 6 samples (1 *RAD51C*, 1 *CDK12*, 1 *FANCF*, and 3 *RAD50*) were considered to be methylation silenced.

b) Genomic scar signatures of methylation samples.
The mean HRD score in the CCLE data was 28.9 and the mean signature 3 was 57.2, indicating that the genomic scar signatures of methylation cases in HR-related genes other than BRCA1 were not significantly elevated.

Figure S3) Association between HR-related gene alterations excluding BRCA1/2 and drug sensitivity

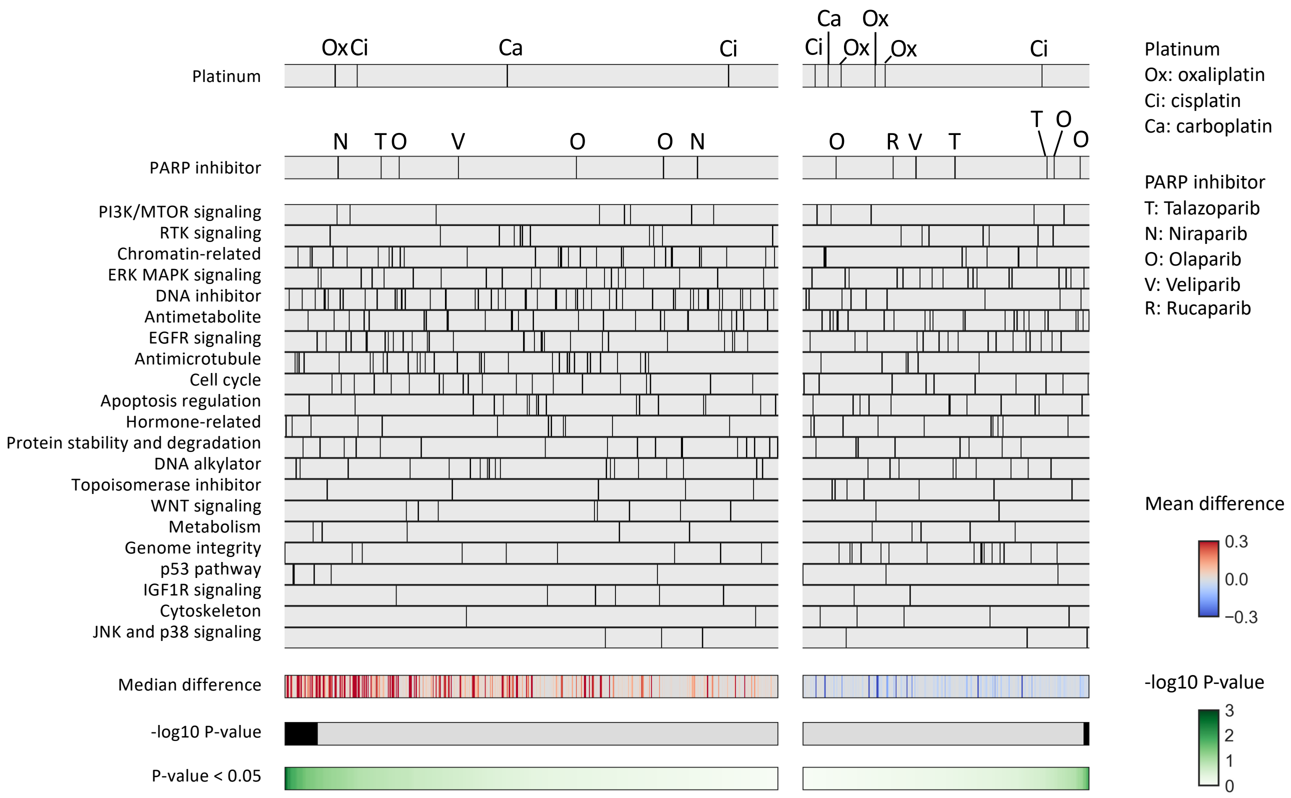

Comparison of the area under the drug-response curve (AUC) between cell lines with HR-related gene alterations excluding *BRCA1/2* (n=57) and no HR-related gene mutations (n=872) was performed.

The drugs were divided into left and right panels based on positive and negative median differences, and further ordered based on the lowest and highest p-values (Mann-Whitney test), respectively.

No platinum and PARP inhibitor assays showed significant correlations with HR-related gene alterations.

Figure S4) Analyses in breast (n=54) and ovarian (n=62) cell lines in the CCLE dataset

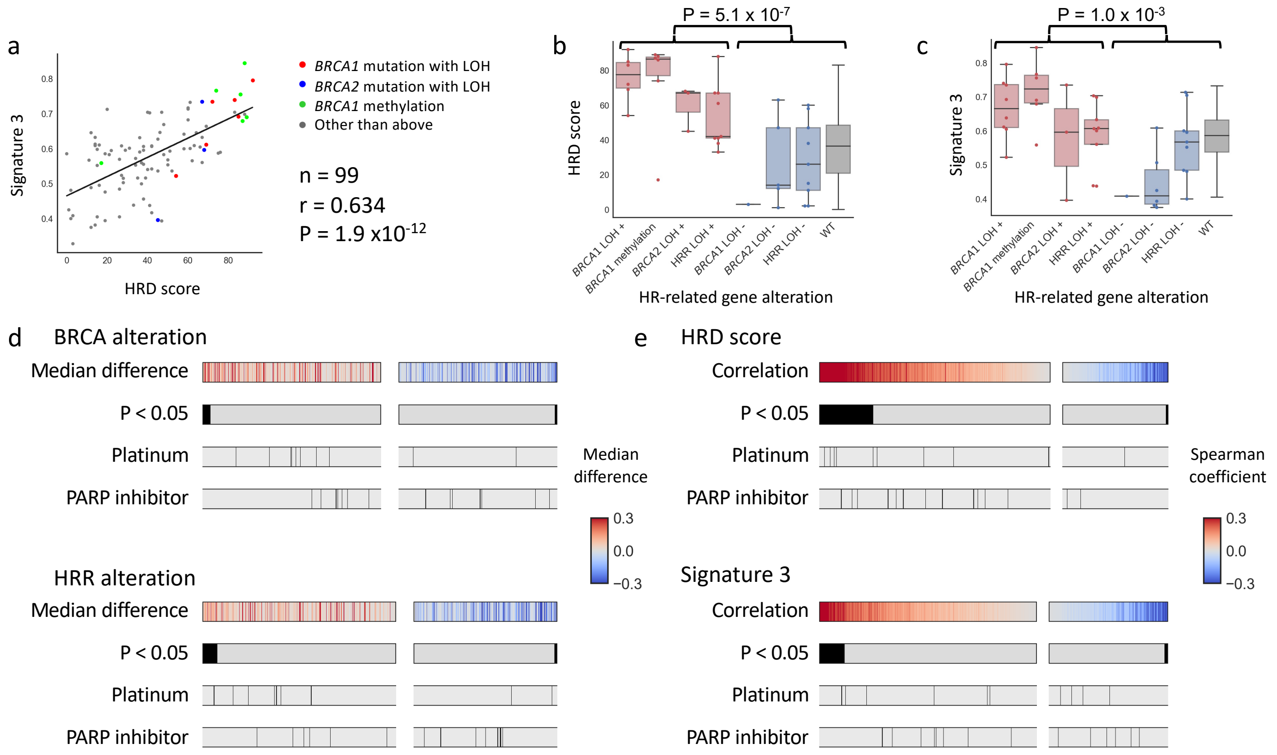

a) Correlation between HRD score and mutational signature 3 and distribution of BRCA1/2 alterations.
HRD scores and mutational signature 3 values were positively correlated.

b) Association between HR-related gene alterations with locus-specific LOH and HRD score.
HRD scores were significantly higher in samples with HR-related gene alterations the those without (Mann-Whitney test, $P = 5.1 \times 10^{-7}$)
HRR LOH +/-: samples with HR-related gene mutations other than BRCA1/2 with/without the locus-specific LOH. WT; samples without any HR-related mutations.

c) Association between HR-related gene alterations with locus-specific LOH and mutational signature 3.
Signature 3 were significantly higher in samples with HR-related gene alterations the those without (Mann-Whitney test, $P = 1.0 \times 10^{-3}$)

d) Association between drug sensitivity and BRCA1/2 alterations (upper) or HR-related gene alterations.
Comparison of the AUCs between cell lines with BRCA1/2 alterations (n=16) or HR-related gene alterations (n=25) vs no HR-related gene mutations (n=83) was performed. The drugs were divided into left and right panels based on positive and negative median differences, and further ordered based on the lowest and highest p-values (Mann-Whitney test), respectively.

e) Spearman's correlation analysis between the drug-response curve (AUC) vs HRD score (upper) or signature 3 (lower).
The drugs were divided into left and right panels based on positive and negative median differences, and further ordered based on the lowest and highest p-values, respectively.

Figure S5) The distributions of HRD scores and mutational signature 3 between CCLE and CLP datasets

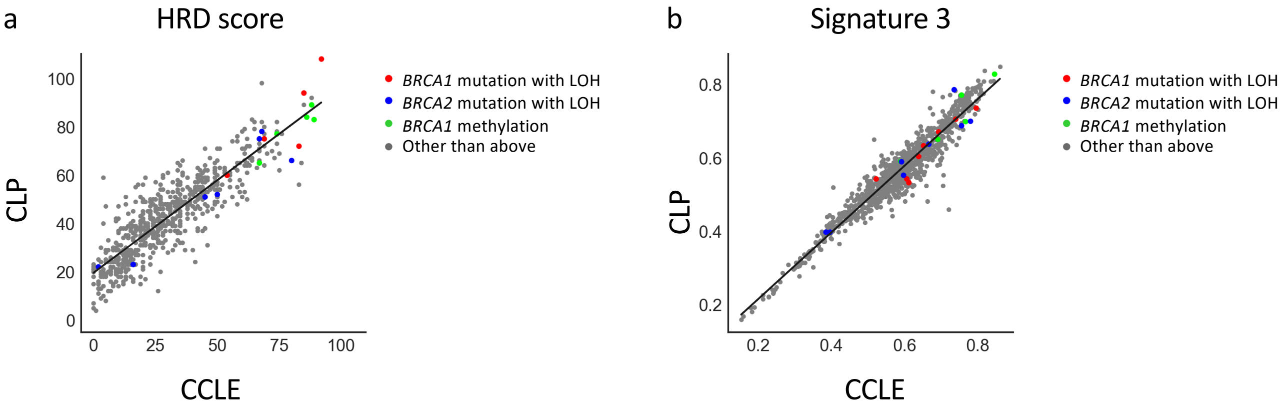

(a)
n = 636
rS = 0.87
$P = 9.8 \times 10^{-197}$

(b)
n = 941
rS = 0.96
$P < 1.0 \times 10^{-199}$

The distribution of HRD scores (a) and mutational signature 3 values (b). Identical cell lines in the two data sets showed similar values to each other.

Figure S6) Association between BRCA1/2 alterations and drug sensitivity in the CLP dataset

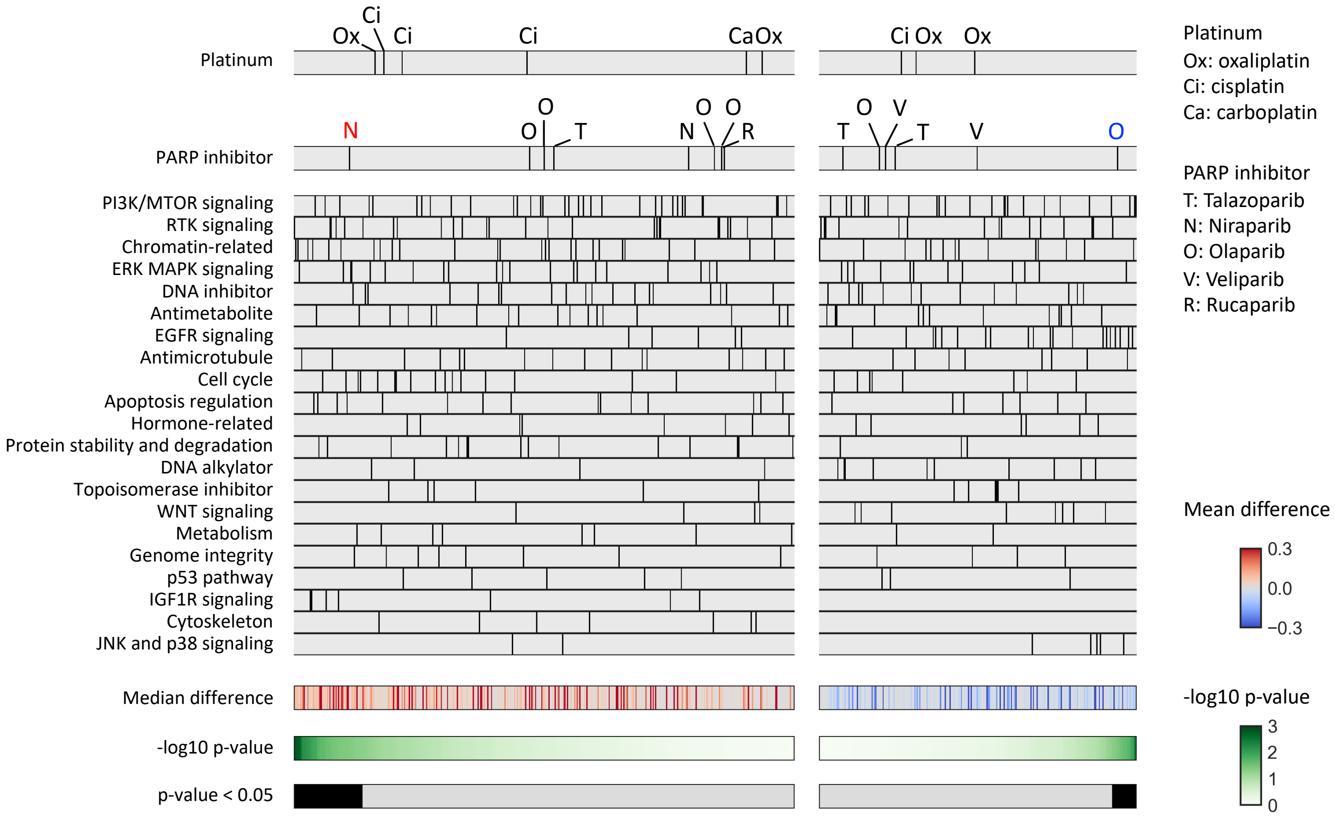

Comparison of the area under the drug-response curve (AUC) between cell lines with BRCA1/2 alterations (n=18) and no HR-related gene mutations (n=774) was performed for each of a total of 669 assays.
The drugs were divided into left and right panels based on positive and negative median differences, and further ordered based on the lowest and highest p-values (Mann-Whitney test), respectively.
Platinum agents (shown in the top panel) and PARP inhibitors (shown in the second panel) exhibiting positive or negative differences (unadjusted P value < 0.05) between the two groups are highlighted in red and blue font, respectively.

Figure S7) Association between HR-related gene alterations and drug sensitivity in the CLP dataset

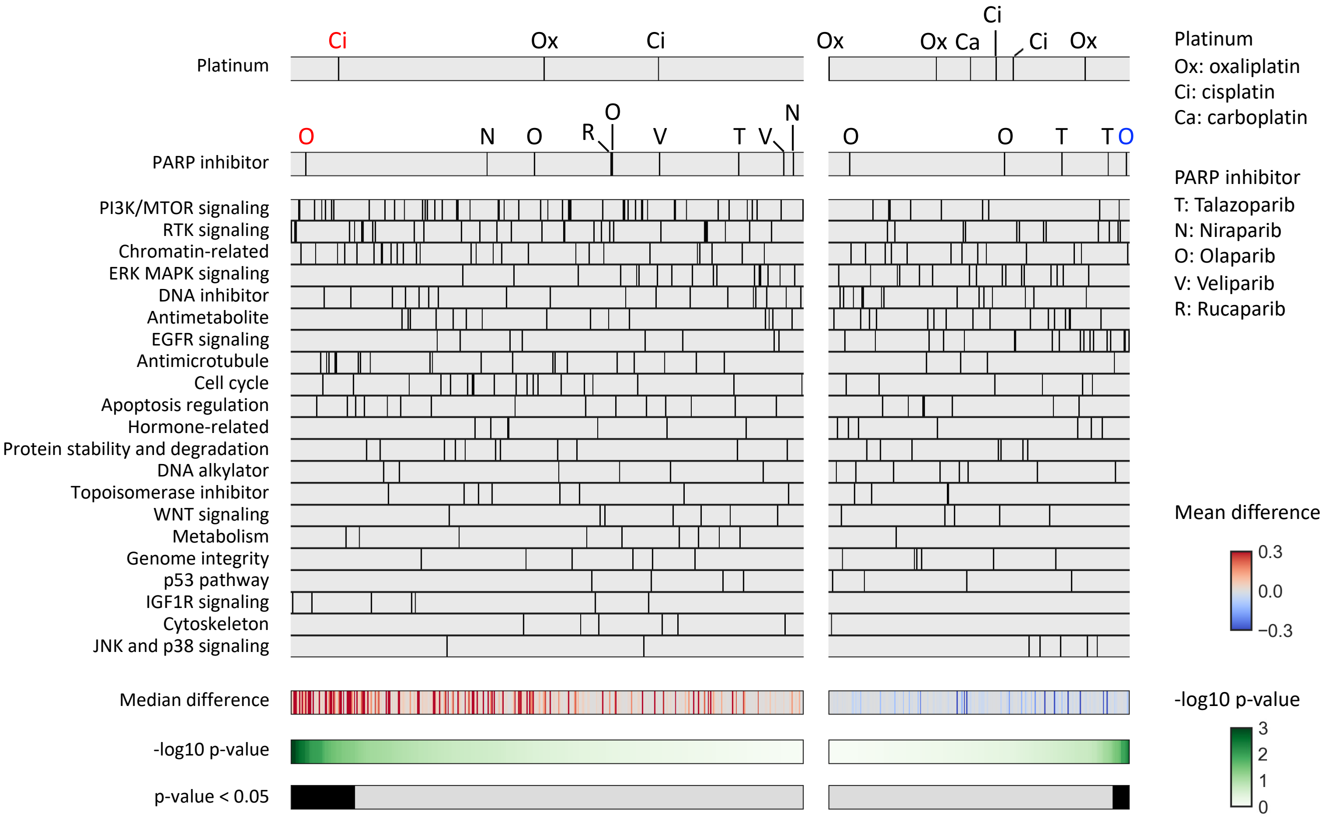

Comparison of the area under the drug-response curve (AUC) between cell lines with HR-related gene alterations, including BRCA1/2, (n=50) and no HR-related gene mutations (n=774) was performed for each of a total of 669 assays.
The drugs were divided into left and right panels based on positive and negative median differences, and further ordered based on the lowest and highest p-values (Mann-Whitney test), respectively.
Platinum agents (shown in the top panel) and PARP inhibitors (shown in the second panel) exhibiting positive or negative differences (unadjusted P value) between the two groups are highlighted in red and blue font, respectively.

Figure S8) Correlations between HRD status vs genomic scar scores in the CLP dataset

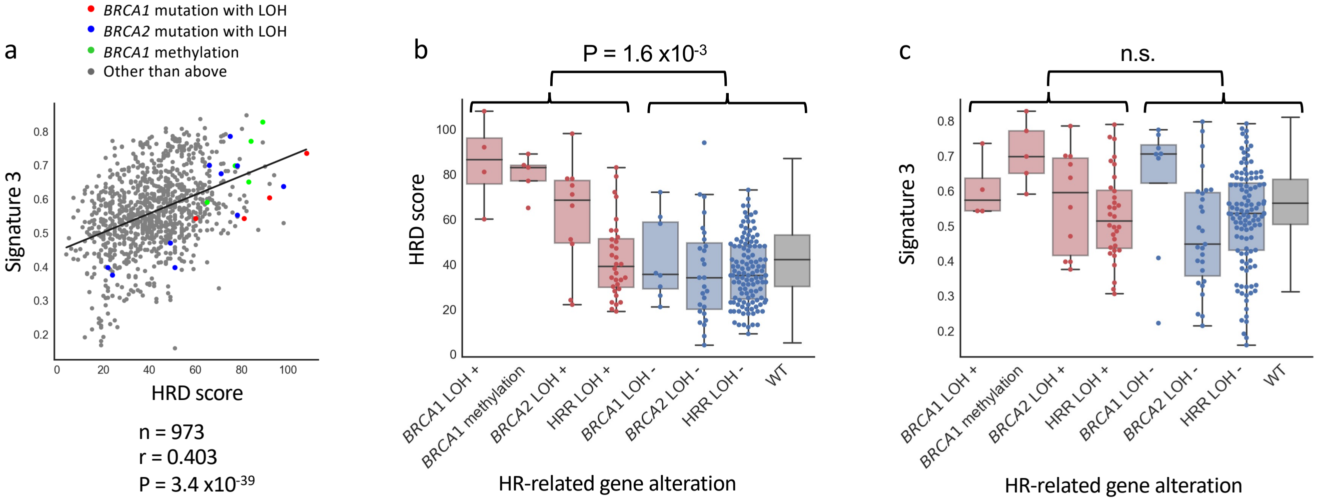

a) Correlation between HRD score and mutational signature 3 and distribution of BRCA1/2 alterations
   HRD scores and mutational signature 3 values were positively correlated.
b) Association between HR-related gene alterations with locus-specific LOH and HRD score.
   HRD scores were significantly higher in samples with HR-related gene alterations the those without (Mann-Whitney test, P = 1.6 x10^{-3}).
c) Association between HR-related gene alterations with locus-specific LOH and mutational signature 3.
   Mutational signature 3 were not significantly different between samples with HR-related gene alterations the those without (Mann-Whitney test, P > 0.05).

Figure S9) Association between HRD score and drug sensitivity in the CLP dataset

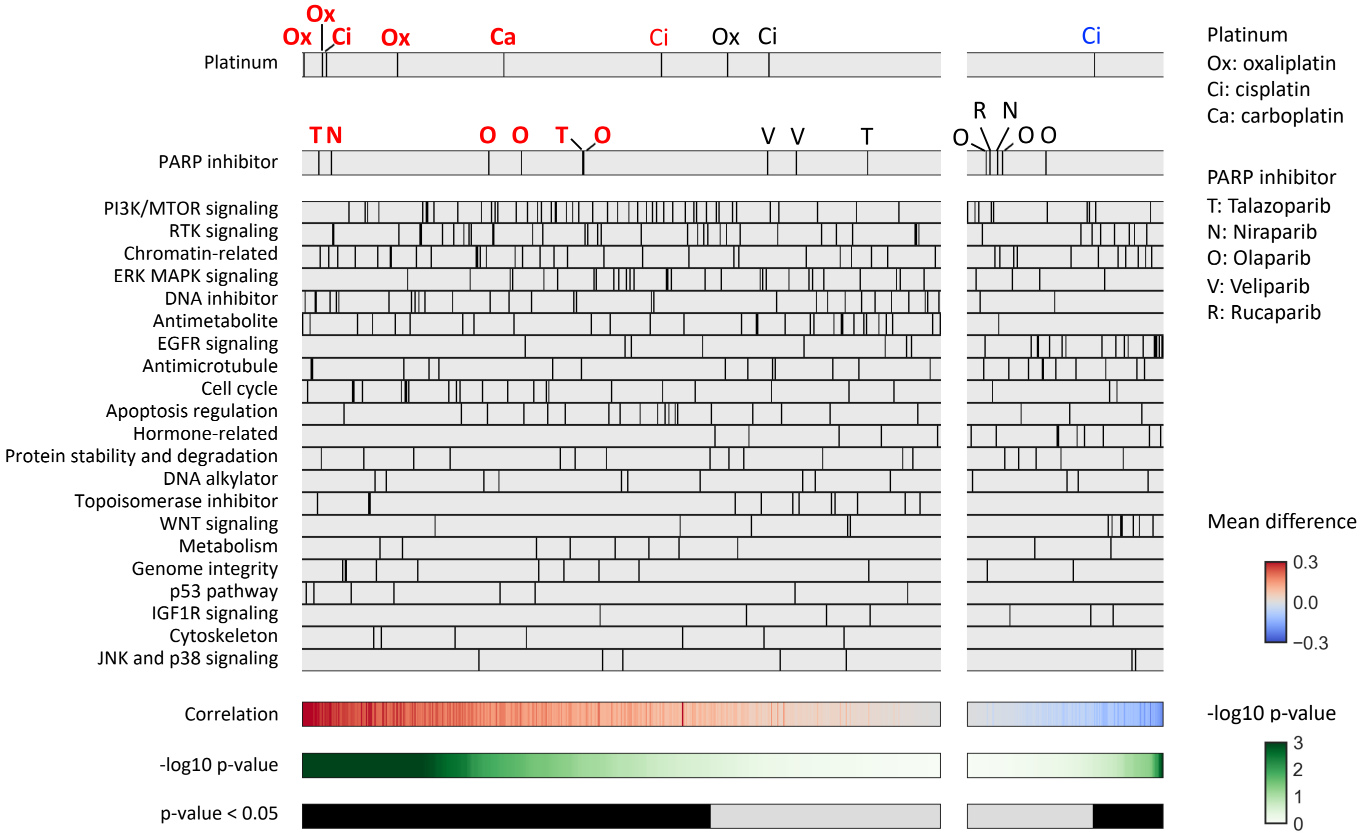

Spearman's correlation between HRD score and the area under the drug-response curve (AUC) was analyzed for each of a total of 669 assays.
The drugs were divided into left and right panels based on positive and negative median differences, and further ordered based on the lowest and highest p-values, respectively. Platinum agents (shown in the top panel) and PARP inhibitors (shown in the second panel) exhibiting positive or negative differences (unadjusted P value < 0.05) between the two groups are highlighted in red or blue font, respectively, and those remained statistically significant (adjusted P value < 0.05) after multiple testing correction are indicated in bold style.

Figure S10) Association between signature 3 and drug sensitivity in the CLP dataset

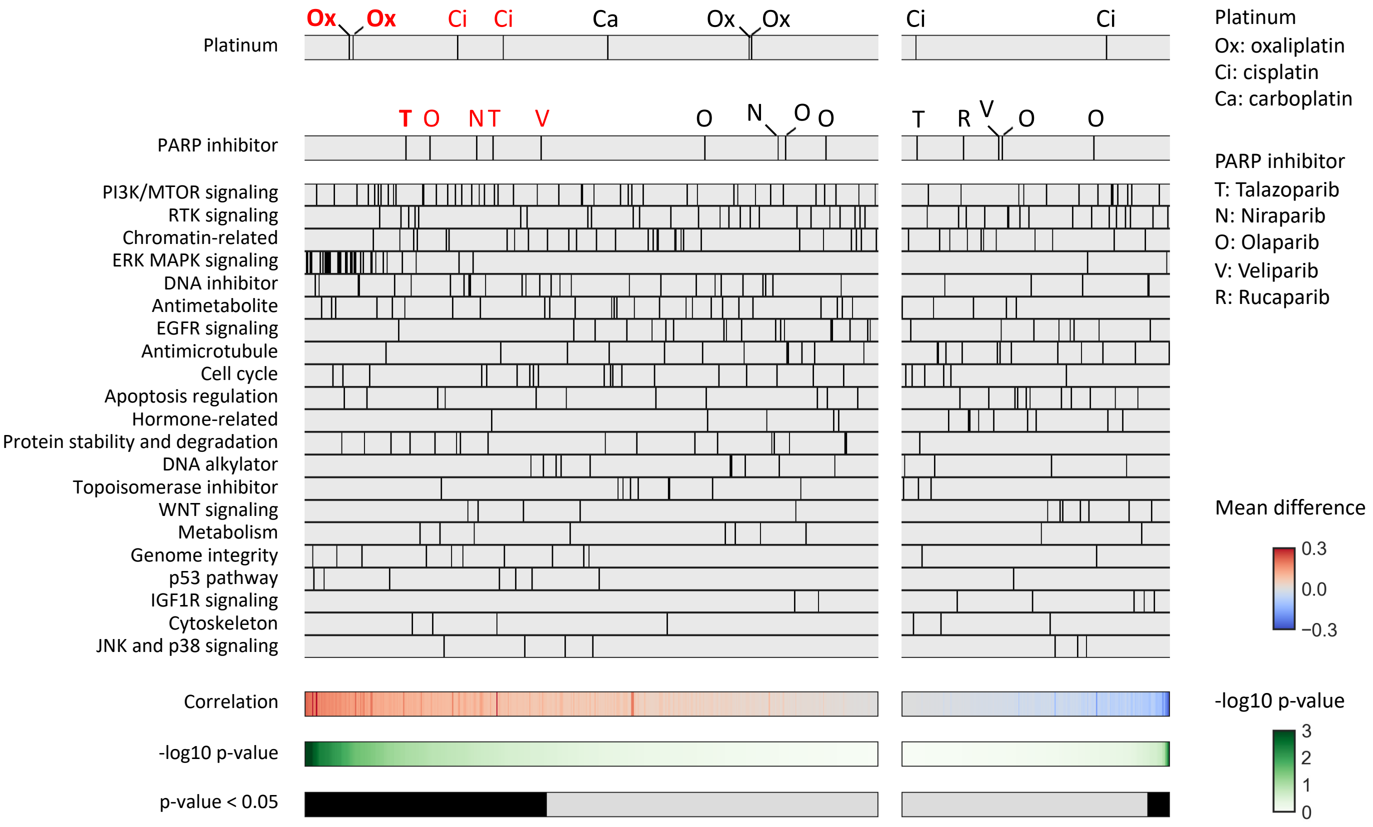

Spearman's correlation between mutational signature 3 value and the area under the drug-response curve (AUC) was analyzed for each of a total of 669 assays.

The drugs were divided into left and right panels based on positive and negative median differences, and further ordered based on the lowest and highest p-values, respectively.

Platinum agents (shown in the top panel) and PARP inhibitors (shown in the second panel) exhibiting positive or negative differences (unadjusted P value < 0.05) between the two groups are highlighted in red or blue font, respectively, and those remained statistically significant (adjusted P value < 0.05) after multiple testing correction are indicated in bold style.

Figure S11) Analysis of methylation silencing in BRCA1

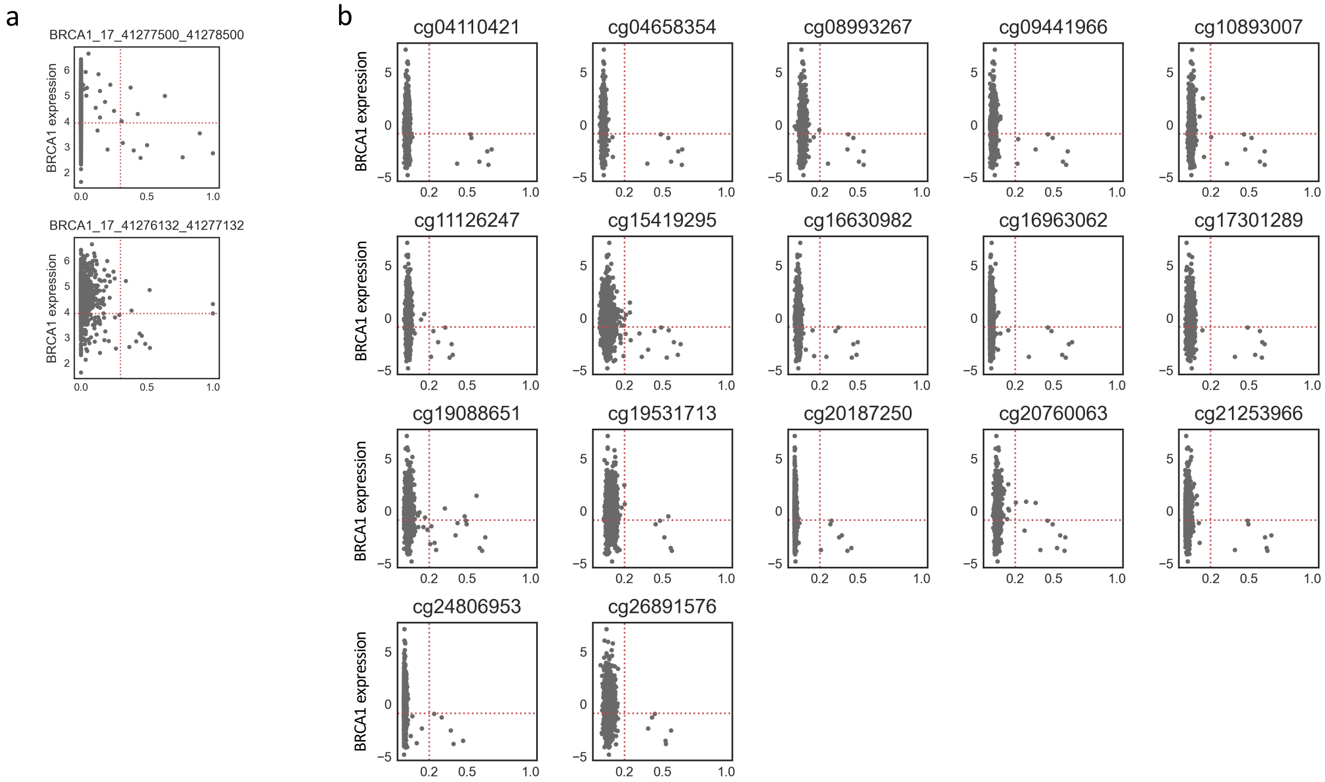

a) CCLE dataset
   Methylation beta values (X-axis) of the two *BRCA1* promoter regions (located 1Kb upstream from the transcription start site) and gene expression (Y-axis) were plotted. Samples with a beta value > 0.3 in two promoter regions and gene expression < 20% were considered to have *BRCA1* methylation.

b) CLP dataset
   Methylation beta values (X-axis) and BRCA1 gene expression (Y-axis) were significant negatively correlated in 17 promoter methylation probes. Samples with beta values > 0.2 and gene expression < 30% for at least 15 of the 17 probes were considered to have BRCA1 methylation.

Table S1) Sources for molecular profiling of cancer cell lines

CCLE project[1], Broad depmap portal[2]: https://depmap.org/portal/download/all				
	Data name	Filename	Related publications	Accession to raw data
Sample information	DepMap Public 22Q2	Sample_info.csv	PMID: 31068700 [3] https://doi.org/10.1038/s41586-019-1186-3	Figshare: https://doi.org/10.6084/m9.figshare.19700056.v2
Gene mutation		CCLE_mutations.csv		
Gene expression		CCLE_expression.csv		
Copy number variations	CCLE2019	CCLE_ABSOLUTE_combined_20181227.xlsx		NCBI BioProject: PRJNA523380
DNA methylation		CCLE_RRBS_TSS1kb_20181022.txt		

COSMIC-Cell Lines Project[4], Sanger Data Downloads: https://cancer.sanger.ac.uk/cell_lines				
	Data name	Filename	Related publications	Accession to raw data
Sample information	QC	QC.xlsx	PMID: 30371878 [5] https://doi.org/10.1093/nar/gky1015 PMID: 27397505 [6] https://doi.org/10.1016/j.cell.2016.06.017	-
Gene mutation	Complete mutation data	CosmicCLP_MutantExport.tsv.gz		European Genome-phenome Archive: EGAS00001000978
Gene expression	Gene Expression	CosmicCLP_RawGeneExpression.tsv.gz		ArrayExpress: E-MTAB-3610
Copy number variations	Copy Number Data	cell_lines_copy_number.csv		European Genome-phenome Archive: EGAS00001000978
DNA methylation	GSE68379	GSE68379_Matrix.processed.txt.gz		NCBI BioProject: PRJNA282584

Reference list:

1. Nature. 2012;483(7391):603–7. doi:10.1038/nature1100
2. Cell. 2017 Jul 27;170(3):564-576.e16. doi: 10.1016/j.cell.2017.06.010
3. Nature. 2019 May;569(7757):503-508. doi: 10.1038/s41586-019-1186-3
4. Nucleic Acids Res. 2024 Jan 5;52(D1):D1210-D1217. doi: 10.1093/nar/gkad986.
5. Nucleic Acids Res. 2019 Jan 8;47(D1):D941-D947. doi: 10.1093/nar/gky1015
6. Cell. 2016 Jul 28;166(3):740-754. doi: 10.1016/j.cell.2016.06.017

Table S2) Sources of drug response data

Study	Link, URL, Data name	Drug-response AUC file name, column name	Drug annotation file name, column name	Nr of drug x cell	Assay
GDSC1 [7,8]	GDSC Resources Download https://www.cancerrxgene.org/downloads/bulk_download GDSC1-dataset, GDSC2-dataset	GDSC1_fitted_dose_response_25Feb20.xlsx AUC	screened_compounds_rel_8.4.csv, TARGET_PATHWAY	240 X 987	72h drug treatment fluorescence-based assay
GDSC2 [6]		GDSC2_fitted_dose_response_25Feb20.csv AUC		166 X 809	
CTRP1 [9]	CTD2 data portal – The Broad Institute https://www.cancer.gov/ccg/research/functional-genomics/ctd2/data-portal/broad-institute CTRP v1 2013 dataset, CTRP v2 2015 dataset	CTRPv1.0_2013_pub_Cell_154_1151/v10.D3.area_under_conc_curve.txt area_under_curve	CTRPv1.0_2013_pub_Cell_154_1151/CTRPv1.0._INFORMER_SET.xlsx, target_or_activity_of_compound & cpd_status #	90 X 240	72h drug treatment CellTiter-Glo assay
CTRP2 [10]		CTRPv2.0_2015_ctd2_ExpandedDataset/v20.data.curves_post_qc.txt area_under_curve	CTRPv2.0_2015_ctd2_ExpandedDataset/CTRPv2.0._INFORMER_SET.xlsx, target_or_activity_of_compound & cpd_status #	173 X 887	
PRISM [11]	depmap portal https://depmap.org/portal/download/ PRISM Repurposing 19Q4	secondary-screen-dose-response-curve-parameters.csv auc	secondary-screen-dose-response-curve-parameters.csv moa & disease.area $	172 X 481	PRISM assay https://www.theprismlab.org/the-prism-assay/
gCSI [12]	PharmacoDB datasets https://pharmacodb.ca/datasets gCSI	gCSI_2019 (script download using R/PharmacoGx) GR_AOC_published	None	41 X 569	72h drug treatment CellTiter-Glo assay.

From "cpd_status" annotation, those with "clinical, FDA approved" were retained
$ From "disease.area" annotation. those with relation to "oncology, malignancy" were retained

Reference list:

7. Nature. 2012 Mar 28;483(7391):570-5. doi: 10.1038/nature11005.
8. Nucleic Acids Res. 2013 Jan;41(Database issue):D955-61. doi: 10.1093/nar/gks1111.
9. Cell. 2013 Aug 29;154(5):1151-1161. doi: 10.1016/j.cell.2013.08.003
10. Nat Chem Biol. 2016 Feb;12(2):109-16. doi: 10.1038/nchembio.1986.
11. Nat Biotechnol. 2016 Apr;34(4):419-23. doi: 10.1038/nbt.3460
12. Nat Biotechnol. 2017 Jun 7;35(6):500-502. doi: 10.1038/nbt.3882.

Table S3) Drug category annotations for each drug Integrated across datasets (GDSC, CTRP, PRISM, gCSI)

Drug_name_common	PubChemID	Drug_annotation	Drug_annotation_primary
GDSC1_CCT-018159_1170	5327091	Protein stability and degradation	Protein stability and degradation
GDSC1_Talazoparib_1259	44819241	PARP inhibitor	Genome integrity
GDSC1_Trametinib_1372	11707110	ERK MAPK signaling	ERK MAPK signaling
GDSC1_Afatinib_1377	10184653	EGFR signaling	EGFR signaling
GDSC1_Bicalutamide_150	2375	Hormone-related	Hormone-related
GDSC1_Methotrexate_1008	126941	Antimetabolite	DNA replication
GDSC1_Olaparib_1017	23725625	PARP inhibitor	Genome integrity
GDSC1_Bosutinib_1019	5328940	Others	Other, kinases
GDSC1_Axitinib_1021	6450551	RTK signaling	RTK signaling
GDSC1_PHA-665752_6	10461815	RTK signaling	RTK signaling
GDSC1_Midostaurin_153	9829523	Others	Other
GDSC1_PFI-3_1530	78243717	Chromatin-related	Chromatin other
GDSC1_JNK-9L_157	25222038	JNK and p38 signaling	JNK and p38 signaling
GDSC1_Linsitinib_185	11640390	IGF1R signaling	IGF1R signaling
GDSC1_Bleomycin_190	5360373	DNA inhibitor	DNA replication
GDSC1_LFM-A13_192	54676905	Others	Other, kinases
GDSC1_BMS-345541_203	9813758	Others	Other, kinases
GDSC1_Ruxolitinib_206	25126798	Others	Other, kinases
GDSC1_AS601245_207	10109823	JNK and p38 signaling	JNK and p38 signaling
GDSC1_AS605240_224	5289247	PI3K/MTOR signaling	PI3K/MTOR signaling
GDSC1_XMD8-85_106	46844147	Others	Other
GDSC1_XMD8-92_1164	46843772	ERK MAPK signaling	ERK MAPK signaling
GDSC1_KIN001-260_290	10451420	DNA inhibitor	DNA replication
GDSC1_NVP-BHG712_295	16747388	Others	Other, kinases
GDSC1_GSK690693_326	16725726	PI3K/MTOR signaling	PI3K/MTOR signaling
GDSC1_KIN001-270_345	66577006	Others	Other
GDSC1_AZD8055_1059	25262965	PI3K/MTOR signaling	PI3K/MTOR signaling
GDSC1_AZD6482_1066	44137675	PI3K/MTOR signaling	PI3K/MTOR signaling
GDSC1_UNC0638_1236	46224516	Chromatin-related	Chromatin histone methylation
GDSC1_Gemcitabine_135	60750	Antimetabolite	DNA replication
GDSC1_Temozolomide_1375	5394	DNA inhibitor	DNA replication
GDSC1_Vinorelbine_140	5311497	Antimicrotubule	Mitosis
GDSC1_Selumetinib_1498	10127622	ERK MAPK signaling	ERK MAPK signaling
GDSC1_Bicalutamide_1502	2375	Hormone-related	Hormone-related
GDSC1_AICA Ribonucleotide_1001	65110	Metabolism	Metabolism
GDSC1_Docetaxel_1007	148124	Antimicrotubule	Mitosis
GDSC1_Vorinostat_1012	5311	Chromatin-related	Chromatin histone acetylation
GDSC1_Temsirolimus_1016	6918289	PI3K/MTOR signaling	PI3K/MTOR signaling
GDSC1_A-770041_55	9549184	Others	Other, kinases
GDSC1_Pyrimethamine_71	4993	Antimetabolite	Other
GDSC1_ZM447439_1050	9914412	Others	Mitosis
GDSC1_OSU-03012_167	10027278	Metabolism	Metabolism
GDSC1_IPA-3_176	521106	Cytoskeleton	Cytoskeleton
GDSC1_BAY-61-3606_178	10200390	Others	Other, kinases
GDSC1_Pazopanib_199	10113978	RTK signaling	RTK signaling
GDSC1_Ispinesib Mesylate_208	6450816	Others	Mitosis
GDSC1_Enzastaurin_229	176167	Others	Other, kinases
GDSC1_CAY10603_276	24951314	Chromatin-related	Chromatin histone acetylation
GDSC1_Linifanib_277	11485656	RTK signaling	RTK signaling
GDSC1_PIK-93_303	6852167	PI3K/MTOR signaling	PI3K/MTOR signaling
GDSC1_Crizotinib_37	11626560	RTK signaling	RTK signaling
GDSC1_Avagacestat_1072	46883536	Others	Other
GDSC1_Cetuximab_1114	85668777	EGFR signaling	EGFR signaling
GDSC1_CHIR-99021_1241	9956119	WNT signaling	WNT signaling
GDSC1_Daporinad_1248	6914657	Metabolism	Metabolism
GDSC1_XAV939_1268	2726824	WNT signaling	WNT signaling
GDSC1_Olaparib_1495	23725625	PARP inhibitor	Genome integrity
GDSC1_Vinblastine_1004	6710780	Antimicrotubule	Mitosis
GDSC1_Gefitinib_1010	123631	EGFR signaling	EGFR signaling
GDSC1_GW441756_1023	9943465	RTK signaling	RTK signaling
GDSC1_SB216763_1025	176158	WNT signaling	WNT signaling
GDSC1_VX-702_1028	10341154	JNK and p38 signaling	JNK and p38 signaling
GDSC1_Motesanib_1029	11667893	RTK signaling	RTK signaling
GDSC1_Elesclomol_1031	300471	Protein stability and degradation	Protein stability and degradation
GDSC1_NU7441_1038	11327430	Genome integrity	Genome integrity
GDSC1_WH-4-023_56	11844351	Others	Other, kinases

GDSC1_JW-7-52-1_83	49836027	PI3K/MTOR signaling	PI3K/MTOR signaling
GDSC1_CHIR-99021_154	9956119	WNT signaling	WNT signaling
GDSC1_Ponatinib_155	24826799	Others	Other, kinases
GDSC1_FTI-277_166	3005532	Others	Other
GDSC1_AKT inhibitor VIII_171	10196499	PI3K/MTOR signaling	PI3K/MTOR signaling
GDSC1_BMS-754807_184	24785538	RTK signaling	RTK signaling
GDSC1_Bryostatin 1_197	5280757	Others	Other
GDSC1_BX-912_222	11754511	Metabolism	Metabolism
GDSC1_Paclitaxel_11	36314	Antimicrotubule	Mitosis
GDSC1_Salubrinal_111	5717801	Others	Other
GDSC1_Lapatinib_119	208908	RTK signaling	RTK signaling
GDSC1_NSC-207895_269	42640	p53 pathway	p53 pathway
GDSC1_WHI-P97_288	3796	Others	Other, kinases
GDSC1_PI-103_302	9884685	Others	Other, kinases
GDSC1_Dasatinib_51	3062316	RTK signaling	RTK signaling
GDSC1_GNF-2_52	5311510	Others	ABL signaling
GDSC1_Pictilisib_1058	17755052	PI3K/MTOR signaling	PI3K/MTOR signaling
GDSC1_EHT-1864_1069	9938202	Cytoskeleton	Cytoskeleton
GDSC1_Serdemetan_1133	11609586	p53 pathway	p53 pathway
GDSC1_SB505124_1194	9858940	RTK signaling	RTK signaling
GDSC1_IOX2_1230	54685215	Others	Other
GDSC1_YK-4-279_1239	44632017	Others	Other
GDSC1_Bleomycin (50 uM)_1378	5460769	DNA inhibitor	DNA replication
GDSC1_AZD7762_1022	11152667	Cell cycle	Cell cycle
GDSC1_Vismodegib_1033	24776445	Others	Other
GDSC1_BX795_1037	10077147	Others	Other, kinases
GDSC1_BI-2536_60	11364421	Cell cycle	Cell cycle
GDSC1_BMS-509744_63	20635522	Others	Other, kinases
GDSC1_MG-132_9	462382	Protein stability and degradation	Protein stability and degradation
GDSC1_JNK Inhibitor VIII_1043	11624601	JNK and p38 signaling	JNK and p38 signaling
GDSC1_DMOG_165	560326	Metabolism	Metabolism
GDSC1_FH535_173	3463933	Others	Other
GDSC1_Obatoclax Mesylate_182	11404337	Apoptosis regulation	Apoptosis regulation
GDSC1_Phenformin_196	8249	Others	Other
GDSC1_Avagacestat_205	46883536	Others	Other
GDSC1_CP724714_255	9874913	EGFR signaling	EGFR signaling
GDSC1_STF-62247_258	704473	Others	Other
GDSC1_AR-42_272	6918848	Chromatin-related	Chromatin histone acetylation
GDSC1_BIX02189_279	46931012	ERK MAPK signaling	ERK MAPK signaling
GDSC1_Pelitinib_282	6445562	EGFR signaling	EGFR signaling
GDSC1_CX-5461_300	25257557	Others	Other
GDSC1_Foretinib_308	42642645	RTK signaling	RTK signaling
GDSC1_Cyclopamine_17	442972	Others	Other
GDSC1_Sunitinib_5	5329102	RTK signaling	RTK signaling
GDSC1_(5Z)-7-Oxozeaenol_1242	9863776	Others	Other, kinases
GDSC1_Piperlongumine_1243	637858	Others	Other
GDSC1_UNC1215_1262	57339144	Chromatin-related	Chromatin other
GDSC1_Doxorubicin_133	31703	DNA inhibitor	DNA replication
GDSC1_NSC-87877_147	5459322	Others	Other
GDSC1_CP466722_152	44551660	Genome integrity	Genome integrity
GDSC1_Erlotinib_1	176870	EGFR signaling	EGFR signaling
GDSC1_Navitoclax_1011	24978538	Apoptosis regulation	Apoptosis regulation
GDSC1_Refametinib_1014	44182295	ERK MAPK signaling	ERK MAPK signaling
GDSC1_Lestaurtinib_1024	126565	Others	Other, kinases
GDSC1_WZ-1-84_59	49821040	Others	Other, kinases
GDSC1_Wee1 Inhibitor_1046	10384072	Cell cycle	Cell cycle
GDSC1_PF-562271_158	11713159	Cytoskeleton	Cytoskeleton
GDSC1_GSK1904529A_202	25124816	RTK signaling	RTK signaling
GDSC1_TAK-715_221	9952773	JNK and p38 signaling	JNK and p38 signaling
GDSC1_Idelalisib_238	11625818	PI3K/MTOR signaling	PI3K/MTOR signaling
GDSC1_Cabozantinib_249	25102847	RTK signaling	RTK signaling
GDSC1_Quizartinib_254	24889392	RTK signaling	RTK signaling
GDSC1_VX-11e_262	11634725	ERK MAPK signaling	ERK MAPK signaling
GDSC1_Omipalisib_283	25167777	PI3K/MTOR signaling	PI3K/MTOR signaling
GDSC1_OSI-930_298	9868037	RTK signaling	RTK signaling
GDSC1_OSI-027_299	44224160	PI3K/MTOR signaling	PI3K/MTOR signaling
GDSC1_Tivozanib_312	9911830	RTK signaling	RTK signaling
GDSC1_SNX-2112_328	24772860	Protein stability and degradation	Protein stability and degradation
GDSC1_T0901317_333	447912	Others	Other
GDSC1_GSK269962A_127	16095342	Cytoskeleton	Cytoskeleton
GDSC1_Imatinib_34	5291	Others	Other, kinases
GDSC1_Saracatinib_38	10302451	Others	Other, kinases

GDSC1_Dactolisib_1057	11977753	PI3K/MTOR signaling	PI3K/MTOR signaling
GDSC1_SB590885_1061	11316960	ERK MAPK signaling	ERK MAPK signaling
GDSC1_Selumetinib_1062	10127622	ERK MAPK signaling	ERK MAPK signaling
GDSC1_BMS-536924_1091	10390396	IGF1R signaling	IGF1R signaling
GDSC1_TW 37_1149	11455910	Apoptosis regulation	Apoptosis regulation
GDSC1_Rucaparib_1175	9931953	PARP inhibitor	Genome integrity
GDSC1_PFI-1_1219	71271629	Chromatin-related	Chromatin other
GDSC1_SGC0946_1264	56962337	Chromatin-related	Chromatin histone methylation
GDSC1_Etoposide_134	36462	DNA inhibitor	DNA replication
GDSC1_Dabrafenib_1373	44462760	ERK MAPK signaling	ERK MAPK signaling
GDSC1_QS11_151	4263900	Others	Other
GDSC1_Pictilisib_1527	17755052	PI3K/MTOR signaling	PI3K/MTOR signaling
GDSC1_Pevonedistat_1529	16720766	Others	Other
GDSC1_Tretinoin_1009	444795	Others	Other
GDSC1_Nilotinib_1013	644241	Others	ABL signaling
GDSC1_Afatinib_1032	10184653	EGFR signaling	EGFR signaling
GDSC1_PLX-4720_1036	24180719	ERK MAPK signaling	ERK MAPK signaling
GDSC1_SL0101_1039	10459196	Others	Other, kinases
GDSC1_Parthenolide_89	7251185	Chromatin-related	Chromatin histone acetylation
GDSC1_HG6-64-1_159	53302361	ERK MAPK signaling	ERK MAPK signaling
GDSC1_Thapsigargin_180	446378	Others	Other
GDSC1_GW-2580_193	11617559	RTK signaling	RTK signaling
GDSC1_AT-7519_219	11338033	Cell cycle	Cell cycle
GDSC1_ZSTK474_223	11647372	PI3K/MTOR signaling	PI3K/MTOR signaling
GDSC1_GSK1070916_226	46885626	Others	Mitosis
GDSC1_AKT inhibitor VIII_228	10196499	PI3K/MTOR signaling	PI3K/MTOR signaling
GDSC1_Sepantronium bromide_268	11178236	Apoptosis regulation	Apoptosis regulation
GDSC1_Alectinib_281	49806720	RTK signaling	RTK signaling
GDSC1_PHA-793887_301	46191454	Cell cycle	Cell cycle
GDSC1_SB52334_304	9967941	RTK signaling	RTK signaling
GDSC1_Fedratinib_306	16722836	Others	Other, kinases
GDSC1_AZ628_29	11676786	ERK MAPK signaling	ERK MAPK signaling
GDSC1_Rapamycin_3	5384616	PI3K/MTOR signaling	PI3K/MTOR signaling
GDSC1_Sorafenib_30	216239	RTK signaling	RTK signaling
GDSC1_S-Trityl-L-cysteine_41	76044	Others	Mitosis
GDSC1_CGP-60474_53	644215	Cell cycle	Cell cycle
GDSC1_CGP-082996_54	24825971	Cell cycle	Cell cycle
GDSC1_CCT007093_1067	2314623	Cell cycle	Cell cycle
GDSC1_Tamoxifen_1199	2733526	Hormone-related	Hormone-related
GDSC1_JQ1_1218	46907787	Chromatin-related	Chromatin other
GDSC1_PLX-4720_1371	24180719	ERK MAPK signaling	ERK MAPK signaling
GDSC1_Refametinib_1526	44182295	ERK MAPK signaling	ERK MAPK signaling
GDSC1_Cisplatin_1005	84691	Platinum	DNA replication
GDSC1_Cytarabine_1006	6253	Antimetabolite	Other
GDSC1_CI-1040_1015	6918454	ERK MAPK signaling	ERK MAPK signaling
GDSC1_Veliparib_1018	11960529	PARP inhibitor	Genome integrity
GDSC1_Lenalidomide_1020	216326	Protein stability and degradation	Protein stability and degradation
GDSC1_Tanespimycin_1026	6505803	Protein stability and degradation	Protein stability and degradation
GDSC1_CMK_64	16663089	Others	Other, kinases
GDSC1_A-443654_86	10172943	PI3K/MTOR signaling	PI3K/MTOR signaling
GDSC1_GW843682X_87	9826308	Cell cycle	Cell cycle
GDSC1_TGX221_94	9907093	PI3K/MTOR signaling	PI3K/MTOR signaling
GDSC1_Nutlin-3a (-)_1047	11433190	p53 pathway	p53 pathway
GDSC1_AZD6482_156	44137675	PI3K/MTOR signaling	PI3K/MTOR signaling
GDSC1_Embelin_172	3218	Apoptosis regulation	Apoptosis regulation
GDSC1_GSK650394_177	25022668	Others	Other, kinases
GDSC1_Luminespib_194	10096043	Protein stability and degradation	Protein stability and degradation
GDSC1_Epothilone B_201	448013	Antimicrotubule	Mitosis
GDSC1_UNC0638_245	46224516	Chromatin-related	Chromatin histone methylation
GDSC1_NG-25_260	53340664	Others	Other, kinases
GDSC1_Bortezomib_104	387447	Protein stability and degradation	Protein stability and degradation
GDSC1_Seliciclib_110	160355	Cell cycle	Cell cycle
GDSC1_Zibotentan_266	9910224	Others	Other
GDSC1_VNLG/124_271	24894414	Chromatin-related	Chromatin histone acetylation
GDSC1_Belinostat_274	6918638	Chromatin-related	Chromatin histone acetylation
GDSC1_I-BET-762_275	46943432	Chromatin-related	Chromatin other
GDSC1_CUDC-101_273	24756910	Others	Other
GDSC1_KIN001-244_287	56965967	Others	Other, kinases
GDSC1_Masitinib_292	10074640	RTK signaling	RTK signaling
GDSC1_Amuvatinib_293	11282283	RTK signaling	RTK signaling
GDSC1_TPCA-1_305	9903786	Others	Other, kinases
GDSC1_Y-39983_309	9810884	Cytoskeleton	Cytoskeleton

GDSC1_YM201636_310	9956222	PI3K/MTOR signaling	PI3K/MTOR signaling
GDSC1_Selisistat_341	5113032	Chromatin-related	Chromatin histone acetylation
GDSC1_Tozasertib_32	5494449	Others	Mitosis
GDSC1_Z-LLNle-CHO_45	16760646	Others	Other
GDSC1_RO-3306_1052	44450571	Cell cycle	Cell cycle
GDSC1_MK-2206_1053	46930998	PI3K/MTOR signaling	PI3K/MTOR signaling
GDSC1_Palbociclib_1054	5330286	Cell cycle	Cell cycle
GDSC1_PD0325901_1060	9826528	ERK MAPK signaling	ERK MAPK signaling
GDSC1_PF-4708671_1129	51371303	PI3K/MTOR signaling	PI3K/MTOR signaling
GDSC1_GSK269962A_1192	16095342	Cytoskeleton	Cytoskeleton
GDSC1_Mitomycin-C_136	5746	DNA alkylator	DNA replication
GDSC1_SN-38_1494	104842	DNA inhibitor	DNA replication
GDSC1_KU-55933_1030	5278396	Genome integrity	Genome integrity
GDSC1_BMS-536924_62	10390396	IGF1R signaling	IGF1R signaling
GDSC1_Entinostat_88	4261	Chromatin-related	Chromatin histone acetylation
GDSC1_GSK319347A_91	11626927	Others	Other, kinases
GDSC1_Doramapimod_1042	156422	JNK and p38 signaling	JNK and p38 signaling
GDSC1_PD173074_1049	1401	RTK signaling	RTK signaling
GDSC1_JQ1_163	46907787	Chromatin-related	Chromatin other
GDSC1_Shikonin_170	5208	Others	Other
GDSC1_PAC-1_175	6753378	Apoptosis regulation	Apoptosis regulation
GDSC1_5-Fluorouracil_179	3385	Antimetabolite	Other
GDSC1_Bexarotene_186	82146	Others	Other
GDSC1_Dacinostat_200	6445533	Chromatin-related	Chromatin histone acetylation
GDSC1_Tipifarnib_204	159324	Others	Other
GDSC1_GSK429286A_230	11373846	Cytoskeleton	Cytoskeleton
GDSC1_FR-180204_263	11493598	ERK MAPK signaling	ERK MAPK signaling
GDSC1_Tubastatin A_265	53394750	Chromatin-related	Chromatin histone acetylation
GDSC1_KIN001-266_291	44143370	DNA inhibitor	DNA replication
GDSC1_MPS-1-IN-1_294	25195352	Others	Mitosis
GDSC1_NVP-TAE684_35	16038120	RTK signaling	RTK signaling
GDSC2_5-Fluorouracil_1073	3385	Antimetabolite	Other
GDSC2_ABT737_1910	11228183	Apoptosis regulation	Apoptosis regulation
GDSC2_Afatinib_1032	10184653	EGFR signaling	EGFR signaling
GDSC2_Afuresertib_1912	46843057	PI3K/MTOR signaling	PI3K/MTOR signaling
GDSC2_AGI-5198_1913	56645356	Metabolism	Metabolism
GDSC2_AGI-6780_1634	71299339	Metabolism	Metabolism
GDSC2_Alisertib_1051	24771867	Others	Mitosis
GDSC2_Alpelisib_1560	56649450	PI3K/MTOR signaling	PI3K/MTOR signaling
GDSC2_AMG-319_2045	68947304	PI3K/MTOR signaling	PI3K/MTOR signaling
GDSC2_Avagacestat_1072	46883536	Others	Other
GDSC2_Axitinib_1021	6450551	RTK signaling	RTK signaling
GDSC2_AZ6102_2109	91663328	WNT signaling	WNT signaling
GDSC2_AZ960_1250	25099184	Others	Other, kinases
GDSC2_AZD3759_1915	78209992	EGFR signaling	EGFR signaling
GDSC2_AZD4547_1786	51039095	RTK signaling	RTK signaling
GDSC2_AZD5363_1916	25227436	Others	Other, kinases
GDSC2_AZD5582_1617	49847690	Apoptosis regulation	Apoptosis regulation
GDSC2_AZD6482_2169	44137675	PI3K/MTOR signaling	PI3K/MTOR signaling
GDSC2_AZD6738_1917	54761306	Genome integrity	Genome integrity
GDSC2_AZD7762_1022	11152667	Cell cycle	Cell cycle
GDSC2_AZD8055_1059	25262965	PI3K/MTOR signaling	PI3K/MTOR signaling
GDSC2_AZD8186_1918	52913813	PI3K/MTOR signaling	PI3K/MTOR signaling
GDSC2_BI-2536_1086	11364421	Cell cycle	Cell cycle
GDSC2_BIBR-1532_2043	9927531	Genome integrity	Genome integrity
GDSC2_Bicalutamide_1502	2375	Hormone-related	Hormone-related
GDSC2_Bleomycin_1812	5460769	DNA inhibitor	DNA replication
GDSC2_Bleomycin (50 uM)_1378	5460769	DNA inhibitor	DNA replication
GDSC2_BMS-345541_1249	9926054	Others	Other
GDSC2_BMS-536924_1091	10390396	IGF1R signaling	IGF1R signaling
GDSC2_Bortezomib_1191	387447	Protein stability and degradation	Protein stability and degradation
GDSC2_Bosutinib_1019	5328940	Others	Other, kinases
GDSC2_Buparlisib_1873	16654980	PI3K/MTOR signaling	PI3K/MTOR signaling
GDSC2_BX795_1037	10077147	Others	Other, kinases
GDSC2_Camptothecin_1003	24360	DNA inhibitor	DNA replication
GDSC2_Carmustine_1807	2578	DNA alkylator	-
GDSC2_CCT-018159_1170	5327091	Protein stability and degradation	Protein stability and degradation
GDSC2_CCT007093_1067	2314623	Cell cycle	Cell cycle
GDSC2_Cediranib_1922	9933475	RTK signaling	RTK signaling
GDSC2_CHIR-99021_2175	9956119	WNT signaling	WNT signaling
GDSC2_Cisplatin_1005	84691	Platinum	DNA replication
GDSC2_CPI-637_2158	131698633	Chromatin-related	Chromatin histone methylation

GDSC2_Crizotinib_1083	11626560	RTK signaling	RTK signaling
GDSC2_Cyclophosphamide_1512	2907	DNA alkylator	DNA replication
GDSC2_Cytarabine_1006	6253	Antimetabolite	Other
GDSC2_CZC24832_1615	42623951	PI3K/MTOR signaling	PI3K/MTOR signaling
GDSC2_Dabrafenib_1373	44462760	ERK MAPK signaling	ERK MAPK signaling
GDSC2_Dacarbazine_1815	2942	Others	Other
GDSC2_Dactinomycin_1911	457193	DNA inhibitor	Other
GDSC2_Dactinomycin_1811	457193	DNA inhibitor	Other
GDSC2_Dactolisib_1057	11977753	PI3K/MTOR signaling	PI3K/MTOR signaling
GDSC2_Daporinad_1248	6914657	Metabolism	Metabolism
GDSC2_Dasatinib_1079	3062316	Others	Other, kinases
GDSC2_Dinaciclib_1180	46926350	Cell cycle	Cell cycle
GDSC2_Docetaxel_1007	148124	Antimicrotubule	Mitosis
GDSC2_Docetaxel_1819	148124	Antimicrotubule	Mitosis
GDSC2_Doramapimod_1042	156422	JNK and p38 signaling	JNK and p38 signaling
GDSC2_EHT-1864_1069	9938202	Cytoskeleton	Cytoskeleton
GDSC2_Elesclomol_1031	300471	Protein stability and degradation	Protein stability and degradation
GDSC2_Entinostat_1593	4261	Chromatin-related	Chromatin histone acetylation
GDSC2_Entospletinib_1630	59473233	Others	Other, kinases
GDSC2_Epirubicin_1511	41867	DNA inhibitor	DNA replication
GDSC2_EPZ004777_1237	56962336	Chromatin-related	Chromatin histone methylation
GDSC2_EPZ5676_1563	57345410	Chromatin-related	Chromatin histone methylation
GDSC2_Erlotinib_1168	176870	EGFR signaling	EGFR signaling
GDSC2_Fludarabine_1813	657237	Antimetabolite	DNA replication
GDSC2_Foretinib_2040	42642645	RTK signaling	RTK signaling
GDSC2_Fulvestrant_1816	104741	Hormone-related	Hormone-related
GDSC2_Fulvestrant_1200	104741	Hormone-related	Hormone-related
GDSC2_GDC0810_1925	56941241	Hormone-related	Hormone-related
GDSC2_Gefitinib_1010	123631	EGFR signaling	EGFR signaling
GDSC2_Gemcitabine_1190	60750	Antimetabolite	DNA replication
GDSC2_glutathione_2439	124886	Others	Other
GDSC2_GNE-317_1926	70676303	PI3K/MTOR signaling	PI3K/MTOR signaling
GDSC2_GSK1904529A_1093	25124816	IGF1R signaling	IGF1R signaling
GDSC2_GSK2578215A_1927	68107965	Others	Other, kinases
GDSC2_GSK2606414_1618	53469448	Metabolism	Metabolism
GDSC2_GSK269962A_1192	16095342	Cytoskeleton	Cytoskeleton
GDSC2_GSK2830371_2359	70983932	Others	Other
GDSC2_GSK343_1627	71268957	Chromatin-related	Chromatin histone methylation
GDSC2_GSK343_2037	71268957	Chromatin-related	Chromatin histone methylation
GDSC2_GSK591_2110	117072552	Chromatin-related	Chromatin histone methylation
GDSC2_GW441756_1023	9943465	RTK signaling	RTK signaling
GDSC2_I-BET-762_1624	46943432	Chromatin-related	Chromatin other
GDSC2_I-BRD9_1928	91668541	Chromatin-related	Chromatin other
GDSC2_Ibrutinib_1799	24821094	Others	Other, kinases
GDSC2_IOX2_2174	54685215	Others	Other
GDSC2_Ipatasertib_1924	24788740	PI3K/MTOR signaling	PI3K/MTOR signaling
GDSC2_Irinotecan_1088	60838	Topoisomerase inhibitor	DNA replication
GDSC2_IWP-2_1576	2155128	WNT signaling	WNT signaling
GDSC2_JNK Inhibitor VIII_1043	11624601	JNK and p38 signaling	JNK and p38 signaling
GDSC2_JQ1_2172	46907787	Chromatin-related	Chromatin other
GDSC2_KRAS (G12C) Inhibitor-12_1855	73555129	ERK MAPK signaling	ERK MAPK signaling
GDSC2_KU-55933_1030	5278396	Genome integrity	Genome integrity
GDSC2_Lapatinib_1558	208908	EGFR signaling	EGFR signaling
GDSC2_LCL161_1557	24737642	Apoptosis regulation	Apoptosis regulation
GDSC2_Leflunomide_1578	3899	Antimetabolite	DNA replication
GDSC2_Lenalidomide_1020	216326	Protein stability and degradation	Protein stability and degradation
GDSC2_Lestaurtinib_1024	126565	Others	Other, kinases
GDSC2_LGK974_1598	46926973	WNT signaling	WNT signaling
GDSC2_Linsitinib_1510	11640390	IGF1R signaling	IGF1R signaling
GDSC2_LJI308_2107	118704762	PI3K/MTOR signaling	PI3K/MTOR signaling
GDSC2_Luminespib_1559	10096043	Protein stability and degradation	Protein stability and degradation
GDSC2_LY2109761_1852	11655119	Others	Other
GDSC2_Methotrexate_1008	126941	Antimetabolite	DNA replication
GDSC2_MG-132_1862	462382	Protein stability and degradation	Protein stability and degradation
GDSC2_MIM1_1996	16241412	Apoptosis regulation	Apoptosis regulation
GDSC2_MIRA-1_1931	227681	p53 pathway	p53 pathway
GDSC2_Mirin_1048	520459	Genome integrity	Genome integrity
GDSC2_Mitoxantrone_1810	4212	DNA inhibitor	DNA replication
GDSC2_MK-1775_1179	24856436	Cell cycle	Cell cycle
GDSC2_MK-2206_1053	46930998	PI3K/MTOR signaling	PI3K/MTOR signaling
GDSC2_MK-8776_2046	16224745	Cell cycle	Cell cycle
GDSC2_ML323_1629	60167849	Protein stability and degradation	Protein stability and degradation

GDSC2_MN-64_1854	2802462	WNT signaling	WNT signaling
GDSC2_Motesanib_1029	11667893	RTK signaling	RTK signaling
GDSC2_Navitoclax_1011	24978538	Apoptosis regulation	Apoptosis regulation
GDSC2_Nelarabine_1814	3011155	DNA inhibitor	DNA replication
GDSC2_Nilotinib_1013	644241	Others	ABL signaling
GDSC2_Niraparib_1177	24958200	PARP inhibitor	Genome integrity
GDSC2_NU7441_1038	11327430	Genome integrity	Genome integrity
GDSC2_Nutlin-3a (-)_1047	11433190	p53 pathway	p53 pathway
GDSC2_NVP-ADW742_1932	9825149	IGF1R signaling	IGF1R signaling
GDSC2_Obatoclax Mesylate_1068	11404337	Apoptosis regulation	Apoptosis regulation
GDSC2_OF-1_1853	35397514	Chromatin-related	Chromatin histone acetylation
GDSC2_Olaparib_1017	23725625	PARP inhibitor	Genome integrity
GDSC2_OSI-027_1594	44224160	PI3K/MTOR signaling	PI3K/MTOR signaling
GDSC2_Osimertinib_1919	71496458	EGFR signaling	EGFR signaling
GDSC2_OTX015_1626	9936746	Chromatin-related	Chromatin other
GDSC2_Oxaliplatin_1089	5310940	Platinum	DNA replication
GDSC2_Oxaliplatin_1806	5310940	Platinum	DNA replication
GDSC2_P22077_1933	46931953	Protein stability and degradation	Protein stability and degradation
GDSC2_Paclitaxel_1080	36314	Antimicrotubule	Mitosis
GDSC2_Palbociclib_1054	5330286	Cell cycle	Cell cycle
GDSC2_PCI-34051_1621	24753719	Chromatin-related	Chromatin histone acetylation
GDSC2_PD0325901_1060	9826528	ERK MAPK signaling	ERK MAPK signaling
GDSC2_PD173074_1049	1401	RTK signaling	RTK signaling
GDSC2_Pevonedistat_1529	16720766	Others	Other
GDSC2_PF-4708671_1129	51371303	PI3K/MTOR signaling	PI3K/MTOR signaling
GDSC2_PFI-1_2173	71271629	Chromatin-related	Chromatin other
GDSC2_PFI3_1620	78243717	Chromatin-related	Chromatin other
GDSC2_Picolinici-acid_1635	1018	Others	Other
GDSC2_Pictilisib_1058	17755052	PI3K/MTOR signaling	PI3K/MTOR signaling
GDSC2_Piperlongumine_1243	637858	Others	Other
GDSC2_PLX-4720_1036	24180719	ERK MAPK signaling	ERK MAPK signaling
GDSC2_PRIMA-1MET_1131	52918385	p53 pathway	p53 pathway
GDSC2_PRT062607_1631	44462758	Others	Other, kinases
GDSC2_Pyridostatin_2044	25227847	DNA inhibitor	DNA replication
GDSC2_Rapamycin_1084	5384616	PI3K/MTOR signaling	PI3K/MTOR signaling
GDSC2_Refametinib_1014	44182295	ERK MAPK signaling	ERK MAPK signaling
GDSC2_Ribociclib_1632	44631912	Cell cycle	Cell cycle
GDSC2_RO-3306_1052	44450571	Cell cycle	Cell cycle
GDSC2_Rucaparib_1175	9931953	PARP inhibitor	Genome integrity
GDSC2_Ruxolitinib_1507	25126798	Others	Other, kinases
GDSC2_RVX-208_1625	24871506	Chromatin-related	Chromatin other
GDSC2_Sabutoclax_1849	46236925	Apoptosis regulation	Apoptosis regulation
GDSC2_Sapitinib_1549	11488320	EGFR signaling	EGFR signaling
GDSC2_Savolitinib_1936	68289010	RTK signaling	RTK signaling
GDSC2_SB216763_1025	176158	WNT signaling	WNT signaling
GDSC2_SB505124_1194	9858940	RTK signaling	RTK signaling
GDSC2_SB590885_1061	11316960	ERK MAPK signaling	ERK MAPK signaling
GDSC2_SCH772984_1564	24866313	ERK MAPK signaling	ERK MAPK signaling
GDSC2_Selumetinib_1736	10127622	ERK MAPK signaling	ERK MAPK signaling
GDSC2_Selumetinib_1062	10127622	ERK MAPK signaling	ERK MAPK signaling
GDSC2_Sepantronium bromide_1941	11178236	Apoptosis regulation	Apoptosis regulation
GDSC2_Serdemetan_1133	11609586	p53 pathway	p53 pathway
GDSC2_SGC0946_2177	56962337	Chromatin-related	Chromatin histone methylation
GDSC2_SL0101_1039	10459196	Others	Other, kinases
GDSC2_SN-38_1494	104842	DNA inhibitor	DNA replication
GDSC2_Sorafenib_1085	216239	Others	Other, kinases
GDSC2_Staurosporine_1034	44259	RTK signaling	RTK signaling
GDSC2_Talazoparib_1259	44819241	PARP inhibitor	Genome integrity
GDSC2_Tamoxifen_1199	2733526	Hormone-related	Hormone-related
GDSC2_Tanespimycin_1026	6505803	Protein stability and degradation	Protein stability and degradation
GDSC2_Taselisib_1561	51001932	PI3K/MTOR signaling	PI3K/MTOR signaling
GDSC2_Telomerase Inhibitor IX_1930	10385095	Genome integrity	Genome integrity
GDSC2_Temozolomide_1375	5394	DNA inhibitor	DNA replication
GDSC2_Temsirolimus_1016	6918289	PI3K/MTOR signaling	PI3K/MTOR signaling
GDSC2_Teniposide_1809	452548	DNA inhibitor	DNA replication
GDSC2_Topotecan_1808	60700	DNA inhibitor	DNA replication
GDSC2_Tozasertib_1096	5494449	Others	Mitosis
GDSC2_Trametinib_1372	11707110	ERK MAPK signaling	ERK MAPK signaling
GDSC2_Tretinoin_1009	444795	Others	Other
GDSC2_TW 37_1149	11455910	Apoptosis regulation	Apoptosis regulation
GDSC2_Ulixertinib_2047	11719003	ERK MAPK signaling	ERK MAPK signaling
GDSC2_Ulixertinib_1908	58641927	ERK MAPK signaling	ERK MAPK signaling

GDSC2_UMI-77_1939	992586	Apoptosis regulation	Apoptosis regulation
GDSC2_Uprosertib_2106	51042438	PI3K/MTOR signaling	PI3K/MTOR signaling
GDSC2_Uprosertib_1553	51042438	PI3K/MTOR signaling	PI3K/MTOR signaling
GDSC2_VE-822_1613	59472121	Genome integrity	Genome integrity
GDSC2_VE821_2111	51000408	Genome integrity	Genome integrity
GDSC2_Veliparib_1018	11960529	PARP inhibitor	Genome integrity
GDSC2_Venetoclax_1909	49846579	Apoptosis regulation	Apoptosis regulation
GDSC2_Vinblastine_1004	6710780	Antimicrotubule	Mitosis
GDSC2_Vincristine_1818	5978	Others	-
GDSC2_Vinorelbine_2048	5311497	Antimicrotubule	Mitosis
GDSC2_Vismodegib_1033	24776445	Others	Other
GDSC2_Vorinostat_1012	5311	Chromatin-related	Chromatin histone acetylation
GDSC2_VX-11e_2096	11634725	ERK MAPK signaling	ERK MAPK signaling
GDSC2_Wee1 Inhibitor_1046	10384072	Cell cycle	Cell cycle
GDSC2_WEHI-539_1997	71297207	Apoptosis regulation	Apoptosis regulation
GDSC2_WIKI4_1940	2984337	WNT signaling	WNT signaling
GDSC2_Wnt-C59_1622	57519544	WNT signaling	WNT signaling
GDSC2_WZ4003_1614	72200024	Others	Other, kinases
GDSC2_XAV939_1268	2726824	WNT signaling	WNT signaling
GDSC2_YK-4-279_1239	44632017	Others	Other
GDSC2_ZM447439_1050	9914412	Others	Mitosis
CTRP1_2-deoxyglucose_347781	439268	Others	inhibitor of glycolysis via hexokinase
CTRP1_ABT-737_411738	11228183	Apoptosis regulation	inhibitor of BCL2, BCL-xL and BCL-W
CTRP1_ABT-751_417983	3035714	Antimicrotubule	inhibitor of beta-tubulin
CTRP1_aspirin_31336	2244	Others	inhibitor of cyclooxygenase 1 (COX1) and 2 (COX2)
CTRP1_ATRA_411870	444795	Others	agonist of retinoid acid (RAR) receptors (RAR?, RAR?, and RAR?)
CTRP1_batimastat_411743	5362422	Others	inhibitor of matrix metallopeptidase 1, 2, 3, 7 and 9
CTRP1_BEZ235_348992	11977753	PI3K/MTOR signaling	inhibitor of PI3K and mTOR kinase activity
CTRP1_BI-2536_347813	11364421	Cell cycle	inhibitor of polo-like kinase 1 (PLK1)
CTRP1_BMS-641988_411807	24768935	Hormone-related	antagonist of androgen receptor
CTRP1_BMS-754807_417981	24785538	RTK signaling	inhibitor of IGF1R
CTRP1_bortezomib_411737	387447	Protein stability and degradation	inhibitor of 26S proteasome
CTRP1_capsaicin_26858	1548943	Others	activator of transient receptor potential cation channel 1 (TRPV1)
CTRP1_carbamazepine_23265	2554	Others	anticonvulsant;inhibitor of voltage-gated sodium channels
CTRP1_ciclosporin_55512	5284373	Others	inhibitor of calcineurin by binding to cyclophilin D
CTRP1_curcumin_467048	969516	Others	natural product;modulator of ROS;modulator of NF-kappa-B signaling
CTRP1_cyanoquinoline 11_417415	17759555	Others	inhibitor of MAP3K8 (TPL2/COT);inhibitor of p-EGFR in cells
CTRP1_CYT-997_616351	11351021	Antimicrotubule	inhibitor of tubulin polymerization
CTRP1_dalcetrapib_411818	6918540	Others	inhibitor of cholesteryl ester transfer protein
CTRP1_darinaparsin_609121	11683005	Others	inducer of ROS
CTRP1_dexamethasone_277354	5743	Others	agonist of glucocorticoid receptor
CTRP1_DG-041_411826	11296282	Others	inhibitor of prostaglandin E receptor 3 (subtype EP3)
CTRP1_dichloroacetic acid_375649	6597	Others	inhibitor of pyruvate dehydrogenase kinase 2
CTRP1_eflornithine_60643	3009	Others	inhibitor of ornithine decarboxylase
CTRP1_etomoxir_411735	9840324	Others	inhibitor of carnitine palmitoyltransferase 1A (liver)
CTRP1_etoposide_50696	36462	DNA inhibitor	inhibitor of topoisomerase II
CTRP1_EX-527_608999	707029	Others	inhibitor of sirtuin 1
CTRP1_fatostatin_122301	1889993	Others	inhibitor of sterol regulatory element binding transcription factor 1 and 2 signaling
CTRP1_FK866_418166	6914657	Metabolism	inhibitor of nicotinamide phosphoribosyltransferase
CTRP1_flutamide_32666	3397	Hormone-related	antagonist of androgen receptor
CTRP1_fosfomycin_351217	446987	Others	inhibitor of pyruvate kinase;inhibitor of MurA (UDP-N-acetylglucosamine Enolpyruvyl Transferase)
CTRP1_fumagillol_411775	222778	Others	natural product;inhibitor of methionyl aminopeptidase 2
CTRP1_gemcitabine_411863	60750	Antimetabolite	inhibitor of DNA replication;inhibitor of ribonucleotide reductase, thymidylate synthetase and cytidine monophosphate (UMP-CMP) kinase
CTRP1_glibenclamide_30713	3488	Others	inhibitor of ATP-dependent K+ channel (Kir6, KATP) and CFTR Cl- channel
CTRP1_GMX-1778_411843	148198	Others	inhibitor of nicotinamide phosphoribosyltransferase
CTRP1_gossypol_25036	3503	Others	inhibitor of lactate dehydrogenase;inhibitor of BCL2 family members
CTRP1_idarubicin_411864	42890	Topoisomerase inhibitor	inhibitor of topoisomerase II
CTRP1_indisulam_411874	216468	Others	inhibitor of carbonic anhydrase isoform IX
CTRP1_irosustat_418037	5287541	Others	inhibitor of steroid sulfatase
CTRP1_itraconazole_63349	3793	Others	anti-fungal agent;inhibitor of hedgehog signaling pathway
CTRP1_kahalalide F_609597	9898671	Others	natural product
CTRP1_lenalidomide_411736	216326	Protein stability and degradation	treatment for myelodysplastic syndrome
CTRP1_linsitinib_417980	11640390	IGF1R signaling	inhibitor of insulin-like growth factor 1 receptor and insulin receptor

CTRP1_lonidamine_345379	39562	Others	inhibitor of hexokinase
CTRP1_losartan_28418	3961	Others	inhibitor of angiotensin II receptor
CTRP1_lovastatin acid_30620	64727	Others	inhibitor of HMG-CoA reductase
CTRP1_maraviroc_466269	3002977	Others	negative allosteric modulator of chemokine (C-C motif) receptor 5
CTRP1_MDV-3100_606365	15951529	Hormone-related	antagonist of androgen receptor
CTRP1_minoxidil_45594	4201	Others	activator of ATP-dependent K+ channels (Kir6, KATP)
CTRP1_mitomycin_50732	5746	DNA alkylator	crosslinks DNA
CTRP1_MK-2206_377381	24964624	Others	inhibitor of AKT
CTRP1_MLN2238_632873	25183872	Others	inhibitor of 20S proteasome at the chymotrypsin-like proteolytic (?5) site
CTRP1_MLN-4924_411809	16720766	Others	inhibitor of Nedd-8 activating enzyme
CTRP1_navitoclax_362342	24978538	Apoptosis regulation	inhibitor of BCL2, BCL-xL and BCL-W
CTRP1_neratinib_418038	9915743	Others	inhibitor of HER2/ERBB2 and EGFR
CTRP1_nifedipine_29818	4485	Others	inhibitor of L-type calcium channel
CTRP1_nimodipine_27791	4497	Others	inhibitor of L-type calcium channel
CTRP1_nutlin-3_411722	216345	Others	inhibitor of P53-HDM2 interaction
CTRP1_NVP-AUY922_418168	53401173	Others	inhibitor of HSP90? and HSP90?
CTRP1_olaparib_411867	23725625	PARP inhibitor	inhibitor of poly (ADP-ribose) polymerase 1 (PARP1) and 2 (PARP2)
CTRP1_orteronel_418169	9796590	Others	inhibitor of 17,20 lyase (Cytochrome P450 17A1)
CTRP1_pemetrexed_411817	446556	Antimetabolite	inhibitor of thymidylate synthetase;inhibitor of dihydrofolate reductase;inhibitor of glycinamide ribonucleotide formyltransferase
CTRP1_penfluridol_607011	33630	Others	antagonist of dopamine receptors;inhibitor of T-type calcium channel
CTRP1_PF-04217903_417978	17754438	Others	inhibitor of c-MET
CTRP1_PK-11195_61780	1345	Others	inhibitor of translocator protein
CTRP1_PX-12_609124	219104	Others	inhibitor of thioredoxin-1
CTRP1_rosiglitazone_33118	77999	Others	activator of PPAR?
CTRP1_rucaparib_628605	9931954	PARP inhibitor	inhibitor of poly (ADP-ribose) polymerase 1
CTRP1_selumetinib_348991	10127622	ERK MAPK signaling	inhibitor of MEK1 and MEK2
CTRP1_serdemetan_418164	60167550	Others	inhibitor of HDM2
CTRP1_sildenafil_411862	5212	Others	inhibitor of phosphodiesterase 5A
CTRP1_simvastatin_27894	54454	Others	inhibitor of HMG-CoA reductase
CTRP1_sirolimus_411830	5284616	PI3K/MTOR signaling	inhibitor of mTOR via FRB domain (in complex with FKBP)
CTRP1_tacrolimus_55450	445643	Others	inhibitor of calcineurin (in complex with FKBP)
CTRP1_tamatinib_417818	11213558	Others	inhibitor of spleen tyrosine kinase
CTRP1_tamoxifen_26972	2733526	Hormone-related	modulator of estrogen receptors
CTRP1_tanespimycin_50134	6505803	Protein stability and degradation	inhibitor of HSP90?
CTRP1_teniposide_27871	452548	DNA inhibitor	inhibitor of topoisomerase II
CTRP1_TG-101348_411808	16722836	Others	inhibitor of Janus kinase 2
CTRP1_tipifarnib-P1_417417	9935249	Others	inhibitor of farnesyltransferase
CTRP1_tivozanib_418170	9911830	RTK signaling	inhibitor of VEGFR
CTRP1_tosedostat_417987	15547703	Others	inhibitor of leucine aminopeptidase 3 (LAP), puromycin-sensitive aminopeptidase (PuSA) and aminopeptidase N
CTRP1_tozasertib_397705	5494449	Others	inhibitor of Aurora Kinase A, B and C
CTRP1_trifluoperazine_26914	5566	Others	antagonist of dopamine receptor D2
CTRP1_triptolide_411720	107985	Others	natural product;modulator of NF-kappa-B signaling
CTRP1_valdecoxib_32372	119607	Others	inhibitor of cyclooxygenase-2 (COX2)
CTRP1_veliparib_606034	11960529	PARP inhibitor	inhibitor of poly (ADP-ribose) polymerase 1 (PARP1) and 2 (PARP2)
CTRP1_vismodegib_397704	24776445	Others	inhibitor of smoothened receptor
CTRP1_vorinostat_56554	5311	Chromatin-related	inhibitor of HDAC1, HDAC2, HDAC3, HDAC6, HDAC8
CTRP1_YM-155_417979	10126189	Others	inhibitor of survivin expression
CTRP1_zileuton_346974	60490	Others	inhibitor of 5-lipoxygenase
CTRP2_tretinoin_23151	444795	Others	agonist of retinoid acid receptors
CTRP2_thalidomide_24197	5426	Others	immunomodulatory drug; binder of cereblon
CTRP2_gossypol_25036	3503	Others	inhibitor of lactate dehydrogenase; inhibitor of BCL2 family members
CTRP2_chlorambucil_25334	2708	DNA alkylator	DNA alkylator
CTRP2_fluorouracil_25344	3385	Antimetabolite	pyrimidine analog; inhibitor of thymidylate synthase
CTRP2_cimetidine_26870	2756	Others	inhibitor of histidine receptor H2
CTRP2_azacitidine_26874	9444	Others	inhibitor of DNA methyltransferase
CTRP2_trifluoperazine_26914	5566	Others	antagonist of dopamine receptor D2
CTRP2_paclitaxel_26956	36314	Antimicrotubule	inhibitor of microtubule assembly
CTRP2_tamoxifen_26972	25010734	Hormone-related	modulator of estrogen receptors
CTRP2_carboplatin_26979	38904	Platinum	inducer of DNA damage
CTRP2_teniposide_27871	452548	DNA inhibitor	inhibitor of topoisomerase II
CTRP2_sildenafil_27872	5212	Others	inhibitor of phosphodiesterase 5A
CTRP2_simvastatin_27894	54454	Others	inhibitor of HMG-CoA reductase
CTRP2_procarbazine_28183	4915	DNA inhibitor	inducer of DNA damage

CTRP2_curcumin_28452	969516	Others	natural product; modulator of ROS; modulator of NF-kappa-B signaling
CTRP2_ciclopirox_28784	2749	Others	substituted pyridone antimycotic; inhibitor of the iron-dependent enzyme ribonucleotide reductase
CTRP2_methotrexate_30371	126941	Antimetabolite	inhibitor of dihydrofolate reductase
CTRP2_lovastatin_30620	53232	Others	inhibitor of HMG-CoA reductase
CTRP2_valdecoxib_32372	119607	Others	inhibitor of cyclooxygenase-2 (COX2)
CTRP2_cyclophosphamide_32620	2907	DNA alkylator	DNA alkylator
CTRP2_dacarbazine_32622	5353562	DNA alkylator	DNA alkylator
CTRP2_niclosamide_32653	4477	Others	inhibitor of STAT3 signaling
CTRP2_prochlorperazine_33166	4917	Others	inhibitor of dopamine receptor D2
CTRP2_ifosfamide_35319	3690	DNA alkylator	DNA alkylator
CTRP2_doxorubicin_36599	32874	Topoisomerase inhibitor	inhibitor of topoisomerase II
CTRP2_topotecan_44580	11972519	Topoisomerase inhibitor	inhibitor of topoisomerase I
CTRP2_etoposide_48589	36462	DNA inhibitor	inhibitor of topoisomerase II
CTRP2_tanespimycin_50134	6505803	Protein stability and degradation	inhibitor of HSP90
CTRP2_mitomycin_50732	5746	DNA alkylator	DNA crosslinker
CTRP2_tacrolimus_50737	445643	Others	inhibitor of calcineurin
CTRP2_dasatinib_52882	3062316	Others	inhibitor of SRC, YES1, EPHA2, c-KIT, and LCK
CTRP2_gefitinib_52926	123631	EGFR signaling	inhibitor of EGFR and AKT1
CTRP2_erlotinib_52928	176871	Others	inhibitor of EGFR and HER2
CTRP2_LBH-589_54210	6918837	Chromatin-related	inhibitor of HDAC1, HDAC2, HDAC3, HDAC6, and HDAC8
CTRP2_ciclosporin_55512	6435893	Others	inhibitor of calcineurin by binding to cyclophilin D
CTRP2_vorinostat_56554	5311	Chromatin-related	inhibitor of HDAC1, HDAC2, HDAC3, HDAC6, and HDAC8
CTRP2_sirolimus_56703	5284616	PI3K/MTOR signaling	inhibitor of mTOR
CTRP2_sitagliptin_58442	4369359	Others	inhibitor of dipetidyl peptidase-4
CTRP2_entinostat_60218	4261	Chromatin-related	inhibitor of HDAC1, HDAC2, HDAC3, HDAC6, and HDAC8
CTRP2_belinostat_60225	6918638	Chromatin-related	inhibitor of HDAC1, HDAC2, HDAC3, HDAC6, and HDAC8
CTRP2_vincristine_62602	5978	Others	inhibitor of mictrotubule assembly
CTRP2_cytarabine hydrochloride_62690	5351180	DNA inhibitor	inducer of DNA damage
CTRP2_itraconazole_63349	14179013	Others	anti-fungal agent; inhibitor of hedgehog signaling pathway
CTRP2_dexamethasone_277354	5743	Others	agonist of glucocorticoid receptor
CTRP2_VX-680_340501	5494449	Others	inhibitor of aurora kinases
CTRP2_imatinib_345041	5291	Others	inhibitor of BCR-ABL1 and c-KIT
CTRP2_O-6-benzylguanine_345712	4578	Others	inhibitor of O(6)-alkylguanine DNA alkyltransferases
CTRP2_decitabine_347775	451668	Others	inhibitor of DNA methyltransferase
CTRP2_BI-2536_347813	11364421	Cell cycle	inhibitor of polo-like kinase 1 (PLK1)
CTRP2_axitinib_348990	6450551	RTK signaling	inhibitor of VEGFRs, c-KIT, and PDGFR alpha and beta
CTRP2_selumetinib_348991	10127622	ERK MAPK signaling	inhibitor of MEK1 and MEK2
CTRP2_NVP-BEZ235_348992	11977753	PI3K/MTOR signaling	inhibitor of PI3K and mTOR kinase activity
CTRP2_vandetanib_349002	3081361	Others	inhibitor of VEGFR2 and EGFR
CTRP2_sorafenib_349006	216239	Others	inhibitor of BRAF, CRAF, and VEGFR2
CTRP2_temozolomide_351043	5394	DNA inhibitor	DNA alkylator
CTRP2_navitoclax_362342	24978538	Apoptosis regulation	inhibitor of BCL2, BCL-xL, and BCL-W
CTRP2_bexarotene_374747	82146	Others	inhibitor of retinoid X receptors
CTRP2_nilotinib_374749	644241	Others	inhibitor of ABL1, BCR, and c-KIT
CTRP2_sunitinib_374750	5329102	RTK signaling	inhibitor of VEGFRs, c-KIT, and PDGFR alpha and beta
CTRP2_bendamustine_374755	65628	DNA alkylator	DNA alkylator
CTRP2_omacetaxine mepesuccinate_375219	-1	Others	inhibitor of protein translation by preventing protein elongation
CTRP2_Platin_375395	23939	DNA alkylator	DNA alkylator; organoplatinum reagent
CTRP2_oxaliplatin_375582	24197464	Platinum	DNA alkylator; organoplatinum reagent
CTRP2_MK-2206_377381	46930998	PI3K/MTOR signaling	inhibitor of AKT1
CTRP2_triptolide_411720	107985	Others	natural product; inhibitor of RNA polymerase II
CTRP2_nutlin-3_411722	216345	p53 pathway	inhibitor of p53-MDM2 interaction
CTRP2_bortezomib_411737	387447	Protein stability and degradation	inhibitor of 26S proteasome
CTRP2_ABT-737_411738	11228183	Apoptosis regulation	inhibitor of BCL2, BCL-xL, and BCL-W
CTRP2_TG-101348_411808	16722836	Others	inhibitor of Janus kinase 2
CTRP2_pevonedistat_411809	71729979	Others	inhibitor of Nedd-8 activating enzyme
CTRP2_GMX-1778_411843	-1	Others	inhibitor of nicotinamide phosphoribosyltransferase
CTRP2_gemcitabine_411863	356653	DNA inhibitor	inhibitor of DNA replication; inhibitor of ribonucleotide reductase, thymidylate synthetase, and cytidine monophosphate (UMP-CMP) kinase
CTRP2_olaparib_411867	23725625	PARP inhibitor	inhibitor of poly (ADP-ribose) polymerase 1 and 2
CTRP2_indisulam_411874	216468	Others	inhibitor of carbonic anhydrase isoform IX
CTRP2_cyanoquinoline 11_417415	-1	Others	inhibitor of MAP3K8; inhibitor of phosphorylated EGFR in cells
CTRP2_tipifarnib-P1_417417	159324	Others	inhibitor of farnesyltransferase
CTRP2_tamatinib_417818	11213558	Others	inhibitor of spleen tyrosine kinase
CTRP2_YM-155_417979	11178236	Apoptosis regulation	inhibitor of survivin expression
CTRP2_tosedostat_417987	15547703	Others	inhibitor of leucine aminopeptidase 3 (LAP), puromycin-sensitive aminopeptidase (PuSA), and aminopeptidase N
CTRP2_neratinib_418038	9915743	Others	inhibitor of EGFR and HER2
CTRP2_serdemetan_418164	11609586	p53 pathway	inhibitor of MDM2

CTRP2_daporinad_418166	6914657	Metabolism	inhibitor of nicotinamide phosphoribosyltransferase
CTRP2_MK-1775_418167	24856436	Cell cycle	inhibitor of WEE1
CTRP2_tivozanib_418170	9911830	RTK signaling	inhibitor of VEGFRs
CTRP2_tandutinib_464458	3038522	Others	inhibitor of c-KIT and VEGFR3
CTRP2_fulvestrant_464880	104741	Hormone-related	antagonist of the estrogen receptor
CTRP2_barasertib_601923	11497983	Others	inhibitor of aurora kinase B
CTRP2_linifanib_606033	11485656	RTK signaling	inhibitor of VEGFRs
CTRP2_veliparib_606034	11960529	PARP inhibitor	inhibitor of poly (ADP-ribose) polymerase 1 (PARP1) and 2 (PARP2)
CTRP2_saracatinib_606035	10302451	Others	inhibitor of SRC and ABL1
CTRP2_afatinib_606135	10184653	EGFR signaling	inhibitor of EGFR and HER2
CTRP2_cediranib_606136	9933475	RTK signaling	inhibitor of VEGFRs
CTRP2_canertinib_606138	156414	Others	inhibitor of EGFR and HER2
CTRP2_obatoclax_606142	16681698	Others	inhibitor of MCL1, BCL2, and BCL-xL
CTRP2_masitinib_606143	10074640	RTK signaling	inhibitor of c-KIT, PDGFRA, and PDGFRB
CTRP2_ZSTK474_606144	11647372	PI3K/MTOR signaling	inhibitor of PI3K catalytic subunits beta, delta, and gamma
CTRP2_brivanib_606246	11234052	Others	inhibitor of VEGFR 1/2
CTRP2_SNS-032_606254	3025986	Cell cycle	inhibitor of cyclin-dependent kinases
CTRP2_TG-100-115_606470	10427712	Others	inhibitor of PI3K catalytic subunits delta and gamma
CTRP2_GSK1059615_606471	23582824	PI3K/MTOR signaling	inhibitor of PI3K and mTOR kinase activity
CTRP2_EX-527_608999	5113032	Chromatin-related	inhibitor of sirtuin 1
CTRP2_darinaparsin_609121	11683005	Antimicrotubule	inducer of ROS; inhibitor of microtubule assembly
CTRP2_PX-12_609124	219104	Others	inhibitor of thioredoxin-1
CTRP2_AZD8055_609639	25262965	PI3K/MTOR signaling	inhibitor of mTOR
CTRP2_nintedanib_628601	9809715	Others	inhibitor of c-KIT, VEGFRs, PDGFRs, and FGFRs
CTRP2_crizotinib_628603	54613769	Others	inhibitor of c-MET and ALK
CTRP2_foretinib_628607	42642645	RTK signaling	inhibitor of MET and VEGFR2
CTRP2_regorafenib_628613	11167602	Others	inhibitor of BRAF, RET, KIT, and VEGFR2
CTRP2_OSI-930_628620	9868037	RTK signaling	inhibitor of c-KIT and VEGFR2
CTRP2_MGCD-265_628622	24901704	Others	inhibitor of c-MET and VEGFRs
CTRP2_lenvatinib_628639	9823820	Others	inhibitor of VEGFRs, c-KIT, and PDGFR alpha and beta
CTRP2_MLN2238_632873	25183872	Others	inhibitor of 20S proteasome at the chymotrypsin-like proteolytic (beta-5) site
CTRP2_lapatinib_634309	208908	EGFR signaling	inhibitor of EGFR and HER2
CTRP2_SGX-523_635882	24779724	Others	inhibitor of MET
CTRP2_alisertib_636711	24771867	Others	inhibitor of aurora kinases A and B
CTRP2_AZD6482_639390	44137675	PI3K/MTOR signaling	inhibitor of PI3K catalytic subunits beta and delta
CTRP2_ruxolitinib_639450	25126798	Others	inhibitor of Janus kinases 1 and 2
CTRP2_bleomycin A2_639531	54608728	DNA inhibitor	inducer of DNA damage
CTRP2_GDC-0941_639759	17755052	PI3K/MTOR signaling	inhibitor of PI3K kinase activity
CTRP2_PHA-793887_640007	46191454	Cell cycle	inhibitor of cyclin-dependent kinases
CTRP2_quizartinib_640011	24889392	RTK signaling	inhibtor of VEGFR3
CTRP2_fingolimod_640157	107970	Others	inhibitor of sphingosine 1-phosphate receptor
CTRP2_pazopanib_640265	11525740	Others	inhibitor of VEGFRs, c-KIT, and PDGFRB
CTRP2_PLX-4032_649420	42611257	Others	inhibitor of BRAF
CTRP2_GSK461364_649862	15983966	Others	inhibitor of polo-like kinase 1 (PLK1)
CTRP2_tigecycline_659993	54686904	Others	analog of tetracycline
CTRP2_etomoxir_660082	123823	Others	inhibitor of carnitine palmitoyltransferase
CTRP2_vorapaxar_660136	10077130	Others	antagonist of proteinase-activated receptor 1 (PAR1)
CTRP2_lomeguatrib_660207	3025944	Others	inhibitor of methylguanine-DNA methyltransferase
CTRP2_SB-743921_660238	9936388	Others	inhibitor of kinesin 11
CTRP2_clofarabine_660288	119182	DNA inhibitor	inducer of DNA damage
CTRP2_XL765_660304	49867926	PI3K/MTOR signaling	inhibitor of mTOR and PI3K kinase acitivities
CTRP2_AZD1480_660306	16659841	Others	inhibitor of Janus kinases 1 and 2
CTRP2_bardoxolone methyl_660318	400769	Others	electrophilic inducer of the NFE2L2-KEAP1 pathway
CTRP2_elocalcitol_660319	11396600	Others	agonist of vitamin D receptor
CTRP2_fluvastatin_660322	446155	Others	inhibitor of HMG-CoA reductase
CTRP2_AZD4547_660325	51039095	RTK signaling	inhibitor of fibroblast growth factor receptors
CTRP2_hyperforin_660327	441298	Others	agonist of calcium-permeable ion channels
CTRP2_MK-0752_660332	9803433	Others	inhibitor of gamma-secretase
CTRP2_semagacestat_660341	9843750	Others	inhibitor of gamma-secretase
CTRP2_bosutinib_660346	5328940	Others	inhibitor of SRC and ABL1
CTRP2_temsirolimus_660358	6918289	PI3K/MTOR signaling	inhibitor of mTOR
CTRP2_abiraterone_660363	132971	Others	inhibitor of 17 alpha-hydroxylase and C17,20 lyase
CTRP2_docetaxel_660364	148124	Antimicrotubule	inhibitor of microtubule assembly
CTRP2_nelarabine_660391	3011155	DNA inhibitor	deoxyguanosine analog; inhibitor of DNA synthesis
CTRP2_rigosertib_660397	6918736	Others	inhibitor of polo-like kinase 1; inhibitor of PI3K catalytic subunits alpha and beta
CTRP2_KW-2449_660413	11427553	Others	inhibitor of FLT3 and AURKA
CTRP2_RAF265_660414	11656518	Others	ihibitor of VEGFR2 and BRAF
CTRP2_silmitasertib_660421	24748573	Others	inhibitor of casein kinase 2
CTRP2_momelotinib_660429	25062766	Others	inhibitor of Janus kinases 1 and 2

CTRP2_CAL-101_660430	11625818	PI3K/MTOR signaling	inhibitor of PI3K catalytic subunit delta
CTRP2_OSI-027_660433	44224160	PI3K/MTOR signaling	inhibitor of mTORC1 and mTORC2
CTRP2_AZD7762_660777	11152667	Cell cycle	inhibitor of checkpoint kinases 1 and 2
CTRP2_birinapant_660778	49836020	Others	SMAC mimetic; inhibitor of inhibitor of apoptosis proteins (IAPs)
CTRP2_KX2-391_660779	23635314	Others	peptide mimetic; inhibitor of SRC activity in cells
CTRP2_cabozantinib_660840	25102847	RTK signaling	inhibitor of c-MET, VEGFR2/3, and RET
CTRP2_ibrutinib_660994	24821094	Others	inhibitor of Bruton's tyrosine kinase
CTRP2_sotrastaurin_661032	10296883	Others	inhibitor of protein kinase C beta
CTRP2_COL-3_665914	54678924	Others	analog of tetracycline
CTRP2_ABT-199_666541	49846579	Apoptosis regulation	inhibitor of BCL2
CTRP2_BYL-719_668495	56649450	PI3K/MTOR signaling	inhibitor of PI3K catalytic subunit alpha
CTRP2_GSK2636771_668723	56949517	Others	inhibitor of PI3K catalytic subunit beta
CTRP2_tivantinib_687406	11494412	Antimicrotubule	inhibitor of MET; inhibitor of microtubule assembly
CTRP2_trametinib_687418	11707110	ERK MAPK signaling	inhibitor of MEK1 and MEK2
CTRP2_RO4929097_687577	49867930	Others	inhibitor of gamma-secretase
CTRP2_dinaciclib_687578	46926350	Cell cycle	inhibitor of cyclin-dependent kinases
CTRP2_MLN2480_687579	25161177	Others	inhibitor of RAF kinases
CTRP2_istradefylline_687694	5311037	Others	antagonist of the adenosine A2A receptor
CTRP2_alvocidib_687720	5287969	Cell cycle	inhibitor of cyclin-dependent kinases
CTRP2_dabrafenib_687954	44462760	ERK MAPK signaling	inhibitor of BRAF
CTRP2_AT13387_688229	11955716	Others	inhibitor of HSP90
CTRP2_linsitinib_705300	11640390	IGF1R signaling	inhibitor of insulin-like growth factor 1 receptor and insulin receptor
PRISM_5-fluorouracil_BRD-K24844714-001-24-5	3385	Antimetabolite	thymidylate synthase inhibitor
PRISM_abemaciclib_BRD-K33622447-066-01-9	46220502	Cell cycle	CDK inhibitor
PRISM_abiraterone_BRD-K50071428-001-03-3	132971	Hormone-related	androgen biosynthesis inhibitor
PRISM_altretamine_BRD-K67043667-001-26-4	2123	Antimetabolite	DNA synthesis inhibitor
PRISM_amsacrine_BRD-K68346641-001-01-4	2179	Topoisomerase inhibitor	topoisomerase inhibitor
PRISM_anagrelide_BRD-K62200014-003-10-5	135409400	Others	phosphodiesterase inhibitor
PRISM_anastrozole_BRD-K52172416-001-11-4	2187	Hormone-related	aromatase inhibitor
PRISM_axitinib_BRD-K29905972-001-06-3	6450551	RTK signaling	PDGFR tyrosine kinase receptor inhibitor, VEGFR inhibitor
PRISM_belinostat_BRD-K17743125-001-08-4	6918638	Chromatin-related	HDAC inhibitor
PRISM_bendamustine_BRD-K17068645-003-04-2	65628	DNA inhibitor	DNA inhibitor
PRISM_bexarotene_BRD-K92441787-001-04-1	82146	Others	retinoid receptor agonist
PRISM_bicalutamide_BRD-A29485665-001-12-8	2375	Hormone-related	androgen receptor antagonist
PRISM_bosutinib_BRD-K99964838-001-11-9	5328940	Others	Abl kinase inhibitor, Bcr-Abl kinase inhibitor, src inhibitor
PRISM_busulfan_BRD-K23204545-001-16-4	2478	DNA inhibitor	DNA inhibitor
PRISM_cabazitaxel_BRD-K06858286-001-01-3	9854073	Antimicrotubule	microtubule inhibitor
PRISM_cabozantinib_BRD-K51544265-001-04-2	25102847	RTK signaling	RET tyrosine kinase inhibitor, VEGFR inhibitor
PRISM_capecitabine_BRD-K61192372-001-08-9	60953	Antimetabolite	DNA synthesis inhibitor, thymidylate synthase inhibitor
PRISM_carboplatin_BRD-K90947825-001-02-7	426756	Platinum	DNA alkylating agent, DNA inhibitor
PRISM_carfilzomib_BRD-K15179879-001-03-2	11556711	Protein stability and degradation	proteasome inhibitor
PRISM_carmofur_BRD-K11630072-001-13-2	2577	Antimetabolite	thymidylate synthase inhibitor
PRISM_carmustine_BRD-K36234266-001-09-8	2578	DNA alkylator	DNA alkylating agent, DNA inhibitor
PRISM_chlorambucil_BRD-K29458283-001-29-9	2708	DNA inhibitor	DNA inhibitor
PRISM_cimetidine_BRD-K34157611-001-16-0	2756	Others	histamine receptor antagonist
PRISM_cinacalcet_BRD-K73838513-003-05-5	156419	Others	calcium channel activator
PRISM_cisplatin_BRD-K69172251-001-08-9	24191118	Platinum	DNA alkylating agent, DNA synthesis inhibitor
PRISM_cladribine_BRD-K93034159-001-25-8	20279	Antimetabolite	adenosine deaminase inhibitor, ribonucleotide reductase inhibitor
PRISM_clofarabine_BRD-K34022604-001-06-6	119182	Antimetabolite	ribonucleotide reductase inhibitor
PRISM_cobimetinib_BRD-K03390685-001-01-7	16222096	ERK MAPK signaling	MEK inhibitor
PRISM_cyclophosphamide_BRD-A09722536-002-18-0	2907	DNA alkylator	DNA alkylating agent
PRISM_cyproterone-acetate_BRD-K41141507-001-16-2	9880	Hormone-related	androgen receptor antagonist
PRISM_cytarabine_BRD-K71847383-001-12-5	5351180	Antimetabolite	ribonucleotide reductase inhibitor
PRISM_dasatinib_BRD-K49328571-001-15-0	3062316	Others	Bcr-Abl kinase inhibitor, ephrin inhibitor, KIT inhibitor, PDGFR tyrosine kinase receptor inhibitor, src inhibitor, tyrosine kinase inhibitor
PRISM_doxifluridine_BRD-K58262659-001-09-7	18343	Antimetabolite	thymidylate synthase inhibitor
PRISM_doxorubicin_BRD-K92093830-003-30-8	32874	Topoisomerase inhibitor	topoisomerase inhibitor
PRISM_enzalutamide_BRD-K56851771-001-06-8	15951529	Hormone-related	androgen receptor antagonist
PRISM_epirubicin_BRD-K04548931-003-16-5	41867	DNA inhibitor	topoisomerase inhibitor
PRISM_estramustine_BRD-A70858459-001-01-7	259331	Antimicrotubule	DNA alkylating agent///DNA synthesis inhibitor, microtubule inhibitor
PRISM_estramustine-phosphate_BRD-K00077635-304-01-2	259329	Antimicrotubule	DNA alkylating agent///DNA synthesis inhibitor, microtubule inhibitor
PRISM_etoposide_BRD-K37798499-001-27-2	36462	DNA inhibitor	topoisomerase inhibitor
PRISM_etoposide-phosphate_BRD-K72533376-001-01-2	6918092	Topoisomerase inhibitor	topoisomerase inhibitor
PRISM_exemestane_BRD-K33425534-001-12-5	60198	Hormone-related	aromatase inhibitor
PRISM_floxuridine_BRD-K47832606-001-30-1	5790	Antimetabolite	DNA synthesis inhibitor
PRISM_fludarabine_BRD-K66788707-001-14-3	657237	Antimetabolite	ribonucleotide reductase inhibitor
PRISM_fludarabine-phosphate_BRD-K71106091-001-06-1	30751	Antimetabolite	ribonucleotide reductase inhibitor

PRISM_flutamide_BRD-K28307902-001-21-7	3397	Hormone-related	androgen receptor antagonist
PRISM_formestane_BRD-A26503646-001-16-6	11273	Hormone-related	aromatase inhibitor
PRISM_ftorafur_BRD-K99383816-001-03-5	5386	Antimetabolite	thymidylate synthase inhibitor
PRISM_fulvestrant_BRD-A85667082-001-12-7	104741	Hormone-related	estrogen receptor antagonist
PRISM_gemcitabine_BRD-K15108141-001-06-6	356653	Antimetabolite	ribonucleotide reductase inhibitor
PRISM_gimeracil_BRD-K35687421-001-02-5	54679224	Antimetabolite	dihydropyrimidine dehydrogenase inhibitor
PRISM_hydroxyurea_BRD-K51747290-001-13-1	3657	Antimetabolite	ribonucleotide reductase inhibitor
PRISM_icotinib_BRD-K31698212-001-02-9	22024915	EGFR signaling	EGFR inhibitor
PRISM_idarubicin_BRD-K69650333-003-14-0	42890	Topoisomerase inhibitor	topoisomerase inhibitor
PRISM_idelalisib_BRD-K60866521-001-07-1	11625818	PI3K/MTOR signaling	PI3K inhibitor
PRISM_ifosfamide_BRD-A67097164-001-18-7	3690	DNA alkylator	DNA alkylating agent
PRISM_imiquimod_BRD-K26657438-001-15-2	57469	Others	interferon inducer, toll-like receptor agonist
PRISM_iobenguane_BRD-K43860855-065-11-9	60860	Others	antineoplastic agent
PRISM_ixabepilone_BRD-K03601870-001-01-2	6445540	Antimicrotubule	microtubule stabilizing agent
PRISM_ixazomib_BRD-K78659596-001-03-9	25183872	Protein stability and degradation	proteasome inhibitor
PRISM_ixazomib-citrate_BRD-A66419424-001-02-4	69040311	Protein stability and degradation	proteasome inhibitor
PRISM_lenalidomide_BRD-A17883755-001-06-1	216326	Protein stability and degradation	anticancer agent
PRISM_lenvatinib_BRD-K39974922-001-04-3	9823820	Others	FGFR inhibitor, KIT inhibitor, PDGFR tyrosine kinase receptor inhibitor, VEGFR inhibitor
PRISM_letrozole_BRD-K88789588-001-14-9	3902	Hormone-related	aromatase inhibitor
PRISM_leucovorin_BRD-A75919782-399-02-6	135403648	Others	folate receptor ligand
PRISM_mechlorethamine_BRD-K12829205-003-02-8	5935	DNA inhibitor	DNA inhibitor
PRISM_melphalan_BRD-K87827419-001-16-8	460612	DNA alkylator	DNA alkylating agent, DNA inhibitor
PRISM_mercaptopurine_BRD-K91601245-001-12-0	667490	Antimetabolite	immunosuppressant, protein synthesis inhibitor, purine antagonist
PRISM_methotrexate_BRD-A55424491-001-19-9	126941	Antimetabolite	dihydrofolate reductase inhibitor
PRISM_midostaurin_BRD-K13646352-001-03-8	24202429	Others	FLT3 inhibitor, KIT inhibitor, PKC inhibitor
PRISM_mitomycin-c_BRD-A48237631-001-04-8	5746	DNA alkylator	DNA alkylating agent, DNA synthesis inhibitor
PRISM_mitoxantrone_BRD-K21680192-300-14-4	4212	DNA inhibitor	topoisomerase inhibitor
PRISM_nelarabine_BRD-K84466663-001-05-4	3011155	DNA inhibitor	DNA synthesis inhibitor, T cell inhibitor
PRISM_nilotinib_BRD-K81528515-001-13-8	644241	Others	Abl kinase inhibitor, Bcr-Abl kinase inhibitor
PRISM_nilutamide_BRD-K23566484-001-15-1	4493	Hormone-related	androgen receptor antagonist
PRISM_nimorazole_BRD-K79145628-001-05-5	23009	DNA inhibitor	bacterial DNA inhibitor
PRISM_niraparib_BRD-K54955827-001-02-2	24958200	PARP inhibitor	PARP inhibitor
PRISM_osimertinib_BRD-K42805893-001-04-9	71496458	EGFR signaling	EGFR inhibitor
PRISM_oxaliplatin_BRD-K78960041-001-05-7	24197464	Platinum	DNA inhibitor
PRISM_panobinostat_BRD-K02130563-001-11-4	6918837	Chromatin-related	HDAC inhibitor
PRISM_pentostatin_BRD-K91543828-001-02-0	439693	Antimetabolite	adenosine deaminase inhibitor, ribonucleotide reductase inhibitor
PRISM_pomalidomide_BRD-A12994259-001-02-1	134780	Others	angiogenesis inhibitor, tumor necrosis factor production inhibitor
PRISM_ponatinib_BRD-K44227013-001-06-4	24826799	Others	Bcr-Abl kinase inhibitor, FLT3 inhibitor, PDGFR tyrosine kinase receptor inhibitor
PRISM_pralatrexate_BRD-A74914197-001-02-9	148121	Antimetabolite	dihydrofolate reductase inhibitor
PRISM_raloxifene_BRD-K63828191-003-32-1	5035	Hormone-related	estrogen receptor antagonist, selective estrogen receptor modulator (SERM)
PRISM_raltitrexed_BRD-K89839824-001-05-3	135400182	Antimetabolite	thymidylate synthase inhibitor
PRISM_ribociclib_BRD-K36788280-001-01-2	44631912	Cell cycle	CDK inhibitor
PRISM_romidepsin_BRD-K61397605-001-03-4	5352062	Chromatin-related	HDAC inhibitor
PRISM_ruxolitinib_BRD-K53972329-001-07-0	25126798	Others	JAK inhibitor
PRISM_SN-38_BRD-K89561498-001-01-7	104842	DNA inhibitor	topoisomerase inhibitor
PRISM_sonidegib_BRD-K19796430-001-05-6	24775005	Others	smoothened receptor antagonist
PRISM_streptozotocin_BRD-K34411947-001-12-5	29327	DNA alkylator	DNA alkylating agent
PRISM_talazoparib_BRD-K95142244-001-01-5	135565082	PARP inhibitor	PARP inhibitor
PRISM_tamibarotene_BRD-K36627727-001-05-4	108143	Others	retinoid receptor agonist
PRISM_tamoxifen_BRD-K93754473-048-20-2	25010734	Hormone-related	estrogen receptor antagonist, selective estrogen receptor modulator (SERM)
PRISM_temoporfin_BRD-K63712959-001-01-8	60751	Others	radical formation stimulant
PRISM_temozolomide_BRD-K32107296-001-16-9	5394	DNA inhibitor	DNA alkylating agent
PRISM_temsirolimus_BRD-K42898655-001-01-8	6918289	PI3K/MTOR signaling	mTOR inhibitor
PRISM_teniposide_BRD-A35588707-001-05-5	452548	DNA inhibitor	topoisomerase inhibitor
PRISM_thioguanine_BRD-K49350383-001-14-5	2723601	Antimetabolite	purine antagonist
PRISM_tipiracil_BRD-K11973162-003-01-8	6323266	Others	thymidine phosphorylase inhibitor
PRISM_topotecan_BRD-K55696337-003-24-4	11972519	Topoisomerase inhibitor	topoisomerase inhibitor
PRISM_toremifene_BRD-K51350053-048-16-5	3005573	Hormone-related	estrogen receptor antagonist, selective estrogen receptor modulator (SERM)
PRISM_ubenimex_BRD-K59574735-001-11-8	72172	Others	leukotriene synthesis inhibitor
PRISM_valrubicin_BRD-K72951360-001-01-4	454216	Topoisomerase inhibitor	DNA inhibitor, topoisomerase inhibitor
PRISM_vandetanib_BRD-K77625799-001-07-7	3081361	Others	EGFR inhibitor, RET tyrosine kinase inhibitor, VEGFR inhibitor
PRISM_vinblastine_BRD-K06519765-065-01-6	13342	Antimicrotubule	microtubule inhibitor, tubulin polymerization inhibitor
PRISM_vincristine_BRD-K12251893-065-04-7	5978	Others	tubulin polymerization inhibitor
PRISM_vindesine_BRD-K59753975-001-02-6	40839	Antimicrotubule	tubulin polymerization inhibitor
PRISM_vinflunine_BRD-K64120610-001-01-4	6918295	Antimicrotubule	microtubule inhibitor

PRISM_vinorelbine_BRD-K69280563-001-01-8	25136944	Antimicrotubule	tubulin polymerization inhibitor
PRISM_vismodegib_BRD-K44827188-001-06-0	24776445	Others	hedgehog pathway inhibitor, smoothened receptor antagonist
PRISM_abiraterone-acetate_BRD-K24048528-001-02-5	9821849	Hormone-related	androgen biosynthesis inhibitor
PRISM_afatinib_BRD-K66175015-001-12-4	10184653	EGFR signaling	EGFR inhibitor
PRISM_afatinib_BRD-K66175015-001-09-0	10184653	EGFR signaling	EGFR inhibitor
PRISM_alectinib_BRD-K11267252-001-04-4	49806720	RTK signaling	ALK tyrosine kinase receptor inhibitor
PRISM_alectinib_BRD-K11267252-001-05-1	49806720	RTK signaling	ALK tyrosine kinase receptor inhibitor
PRISM_aminoglutethimide_BRD-A25234499-001-19-1	2145	Others	glucocorticoid receptor antagonist
PRISM_aminoglutethimide_BRD-A25234499-001-18-3	2145	Others	glucocorticoid receptor antagonist
PRISM_azacitidine_BRD-K03406345-001-21-1	9444	Antimetabolite	DNA methyltransferase inhibitor
PRISM_azacitidine_BRD-K03406345-001-27-8	9444	Antimetabolite	DNA methyltransferase inhibitor
PRISM_bortezomib_BRD-K88510285-001-17-8	387447	Protein stability and degradation	NFkB pathway inhibitor, proteasome inhibitor
PRISM_bortezomib_BRD-K88510285-001-11-1	387447	Protein stability and degradation	NFkB pathway inhibitor, proteasome inhibitor
PRISM_brigatinib_BRD-K56981171-001-01-0	68165256	Others	ALK tyrosine kinase receptor inhibitor, EGFR inhibitor
PRISM_brigatinib_BRD-K56981171-001-02-8	68165256	Others	ALK tyrosine kinase receptor inhibitor, EGFR inhibitor
PRISM_crizotinib_BRD-K78431006-001-10-2	54613769	Others	ALK tyrosine kinase receptor inhibitor
PRISM_crizotinib_BRD-K78431006-001-15-1	54613769	Others	ALK tyrosine kinase receptor inhibitor
PRISM_dabrafenib_BRD-K09951645-001-11-8	44462760	ERK MAPK signaling	RAF inhibitor
PRISM_dabrafenib_BRD-K09951645-001-06-8	44462760	ERK MAPK signaling	RAF inhibitor
PRISM_dacarbazine_BRD-K35520305-001-17-7	5353562	DNA alkylator	DNA alkylating agent
PRISM_dacarbazine_BRD-K35520305-001-16-9	5353562	DNA alkylator	DNA alkylating agent
PRISM_daunorubicin_BRD-K43389675-003-20-9	30323	Topoisomerase inhibitor	RNA synthesis inhibitor, topoisomerase inhibitor
PRISM_daunorubicin_BRD-K43389675-001-02-1	30323	Topoisomerase inhibitor	RNA synthesis inhibitor, topoisomerase inhibitor
PRISM_decitabine_BRD-K79254416-001-22-6	451668	Others	DNA methyltransferase inhibitor
PRISM_decitabine_BRD-K79254416-001-21-8	451668	Others	DNA methyltransferase inhibitor
PRISM_docetaxel_BRD-K30577245-341-01-9	148124	Antimicrotubule	tubulin polymerization inhibitor
PRISM_docetaxel_BRD-K30577245-001-05-0	148124	Antimicrotubule	tubulin polymerization inhibitor
PRISM_erlotinib_BRD-K70401845-001-15-7	176871	EGFR signaling	EGFR inhibitor
PRISM_erlotinib_BRD-K70401845-003-09-6	176871	EGFR signaling	EGFR inhibitor
PRISM_everolimus_BRD-K13514097-001-04-6	6442177	PI3K/MTOR signaling	mTOR inhibitor
PRISM_everolimus_BRD-K13514097-001-05-3	6442177	PI3K/MTOR signaling	mTOR inhibitor
PRISM_gefitinib_BRD-K64052750-001-17-5	123631	EGFR signaling	EGFR inhibitor
PRISM_gefitinib_BRD-K64052750-001-22-5	123631	EGFR signaling	EGFR inhibitor
PRISM_ibrutinib_BRD-K70301465-001-02-6	24821094	Others	Bruton's tyrosine kinase (BTK) inhibitor
PRISM_ibrutinib_BRD-K70301465-001-05-9	24821094	Others	Bruton's tyrosine kinase (BTK) inhibitor
PRISM_imatinib_BRD-K92723993-001-17-4	5291	Others	Bcr-Abl kinase inhibitor, KIT inhibitor, PDGFR tyrosine kinase receptor inhibitor
PRISM_imatinib_BRD-K92723993-001-06-7	5291	Others	Bcr-Abl kinase inhibitor, KIT inhibitor, PDGFR tyrosine kinase receptor inhibitor
PRISM_irinotecan_BRD-K08547377-001-04-4	60838	Topoisomerase inhibitor	topoisomerase inhibitor
PRISM_irinotecan_BRD-K08547377-394-03-5	60838	Topoisomerase inhibitor	topoisomerase inhibitor
PRISM_lapatinib_BRD-K19687926-379-07-4	208908	EGFR signaling	EGFR inhibitor
PRISM_lapatinib_BRD-K19687926-001-04-1	208908	EGFR signaling	EGFR inhibitor
PRISM_olaparib_BRD-K02113016-001-19-6	23725625	PARP inhibitor	PARP inhibitor
PRISM_olaparib_BRD-K02113016-001-15-4	23725625	PARP inhibitor	PARP inhibitor
PRISM_paclitaxel_BRD-K62008436-001-23-9	36314	Antimicrotubule	tubulin polymerization inhibitor
PRISM_paclitaxel_BRD-K62008436-001-22-1	36314	Antimicrotubule	tubulin polymerization inhibitor
PRISM_palbociclib_BRD-K51313569-001-07-8	5330286	Cell cycle	CDK inhibitor
PRISM_palbociclib_BRD-K51313569-003-03-3	5330286	Cell cycle	CDK inhibitor
PRISM_pazopanib_BRD-K74514084-003-09-2	11525740	Others	KIT inhibitor, PDGFR tyrosine kinase receptor inhibitor, VEGFR inhibitor
PRISM_pazopanib_BRD-K74514084-001-03-9	11525740	Others	KIT inhibitor, PDGFR tyrosine kinase receptor inhibitor, VEGFR inhibitor
PRISM_regorafenib_BRD-K16730910-001-10-7	11167602	Others	FGFR inhibitor, KIT inhibitor, PDGFR tyrosine kinase receptor inhibitor, RAF inhibitor, RET tyrosine kinase inhibitor, VEGFR inhibitor
PRISM_regorafenib_BRD-K16730910-001-07-3	11167602	Others	FGFR inhibitor, KIT inhibitor, PDGFR tyrosine kinase receptor inhibitor, RAF inhibitor, RET tyrosine kinase inhibitor, VEGFR inhibitor
PRISM_sorafenib_BRD-K23984367-001-07-5	216239	Others	FLT3 inhibitor, KIT inhibitor, PDGFR tyrosine kinase receptor inhibitor, RAF inhibitor, RET tyrosine kinase inhibitor, VEGFR inhibitor
PRISM_sorafenib_BRD-K23984367-075-15-2	216239	Others	FLT3 inhibitor, KIT inhibitor, PDGFR tyrosine kinase receptor inhibitor, RAF inhibitor, RET tyrosine kinase inhibitor, VEGFR inhibitor
PRISM_sunitinib_BRD-K42828737-001-03-3	5329102	RTK signaling	FLT3 inhibitor, KIT inhibitor, PDGFR tyrosine kinase receptor inhibitor, RET tyrosine kinase inhibitor, VEGFR inhibitor
PRISM_sunitinib_BRD-K42828737-044-09-0	5329102	RTK signaling	FLT3 inhibitor, KIT inhibitor, PDGFR tyrosine kinase receptor inhibitor, RET tyrosine kinase inhibitor, VEGFR inhibitor
PRISM_trametinib_BRD-K12343256-001-08-9	11707110	ERK MAPK signaling	MEK inhibitor
PRISM_trametinib_BRD-K12343256-001-14-7	11707110	ERK MAPK signaling	MEK inhibitor
PRISM_vemurafenib_BRD-K56343971-001-14-8	42611257	Others	RAF inhibitor
PRISM_vemurafenib_BRD-K56343971-001-10-6	42611257	Others	RAF inhibitor
PRISM_venetoclax_BRD-K62391742-001-09-7	49846579	Apoptosis regulation	BCL inhibitor

PRISM_venetoclax_BRD-K62391742-001-03-0	49846579	Apoptosis regulation	BCL inhibitor
PRISM_vorinostat_BRD-K81418486-001-47-5	5311	Chromatin-related	HDAC inhibitor
PRISM_vorinostat_BRD-K81418486-001-44-2	5311	Chromatin-related	HDAC inhibitor
gCSI_5-Fluorouracil_CID3385	3385	Antimetabolite	None
gCSI_AZ-628_CID11676786	11676786	ERK MAPK signaling	None
gCSI_Azacitidine_CID9444	9444	Others	None
gCSI_AZD7762_CID11152667	11152667	Cell cycle	None
gCSI_AZD8055_CID25262965	25262965	PI3K/MTOR signaling	None
gCSI_Bortezomib_CID387447	387447	Protein stability and degradation	None
gCSI_Bosutinib_CID5328940	5328940	Others	None
gCSI_Chir-99021_CID9956119	9956119	WNT signaling	None
gCSI_Cisplatin_CID24191118	24191118	Platinum	None
gCSI_Crizotinib_CID54613769	54613769	RTK signaling	None
gCSI_Dabrafenib_CID44462760	44462760	ERK MAPK signaling	None
gCSI_Dasatinib_CID3062316	3062316	Others	None
gCSI_Docetaxel_CID148124	148124	Antimicrotubule	None
gCSI_Erastin_CID11214940	11214940	Others	None
gCSI_Erlotinib_CID176871	176871	EGFR signaling	None
gCSI_Etoposide_CID36462	36462	DNA inhibitor	None
gCSI_Gefitinib_CID123631	123631	EGFR signaling	None
gCSI_Gemcitabine_CID356653	356653	Antimetabolite	None
gCSI_GSK2636771_CID56949517	56949517	Others	None
gCSI_Ibrutinib_CID24821094	24821094	Others	None
gCSI_Idelalisib_CID11625818	11625818	PI3K/MTOR signaling	None
gCSI_JQ1 compound_CID71300862	71300862	Others	None
gCSI_Lapatinib_CID208908	208908	EGFR signaling	None
gCSI_Lenalidomide_CID216326	216326	Protein stability and degradation	None
gCSI_Methotrexate_CID126941	126941	Antimetabolite	None
gCSI_MI-2_CID54765302	54765302	Others	None
gCSI_MK-2206_CID46930998	46930998	PI3K/MTOR signaling	None
gCSI_MLN2480_CID25161177	25161177	Others	None
gCSI_Narciclasine_CID72376	72376	Others	None
gCSI_Nilotinib_CID644241	644241	Others	None
gCSI_NU-7441_CID11327430	11327430	Genome integrity	None
gCSI_Oligomycin A_CID5472285	5472285	Others	None
gCSI_Paclitaxel_CID36314	36314	Antimicrotubule	None
gCSI_Palbociclib_CID5330286	5330286	Cell cycle	None
gCSI_PF-4708671_CID51371303	51371303	PI3K/MTOR signaling	None
gCSI_Pictilisib_CID17755052	17755052	PI3K/MTOR signaling	None
gCSI_PLX4720_CID24180719	24180719	ERK MAPK signaling	None
gCSI_Tanespimycin_CID6505803	6505803	Protein stability and degradation	None
gCSI_TGX221_CID9907093	9907093	PI3K/MTOR signaling	None
gCSI_Tivantinib_CID11494412	11494412	Others	None
gCSI_Vincaleukoblastine_CID241902	241902	Antimicrotubule	None
gCSI_Vincristine_CID5978	5978	Others	None
gCSI_Vorinostat_CID5311	5311	Chromatin-related	None
